# Data showing the lipid conformations and membrane binding behaviors of beta-amyloid fibrils in phase-separated cholesterol-enriched lipid domains with and without glycolipid and oxidized cholesterol from coarse-grained molecular dynamics simulations

**DOI:** 10.1016/j.dib.2020.105496

**Published:** 2020-04-19

**Authors:** Sara Y. Cheng, Yiyi Cao, Marzieh Rouzbehani, Kwan H. Cheng

**Affiliations:** a Department of Physics, University of Texas at Austin, Austin, TX, United States; b Department of Neuroscience, Trinity University, San Antonio, TX, United States; c Department of Physics, Trinity University, San Antonio, TX, United States

**Keywords:** Amyloid fibrils, Lipid rafts, Coarse-grained MD simulations, Protein insertion, Membrane-bound protein orientation, Protein binding kinetics on membranes

## Abstract

The structural conformations of phospholipids and cholesterol in phase-separated lipid domains were determined by surface area, transverse density profile, and lipid acyl chain orientational parameter calculations. Binding kinetics and characterization of membrane-bound states of beta-amyloid fibrils of various sizes (dimer to pentamer), on those lipid domains, were determined using protein residue orientational parameter and fibril-residue-lipid minimum distance analysis methods. The energy of binding and characterization of annular lipid shells surrounding the surface-bound amyloid fibrils were also determined. The calculations described above support the article “Coarse-Grained MD simulations Reveal Diverse Membrane-Bound Conformational States of Beta-Amyloid Fibrils in the Liquid-ordered and Liquid-disordered Regions of Phase-Separated Lipid Rafts Containing Glycolipid, Cholesterol and Oxidized Cholesterol (Cheng et al., 2020 [1])”. The reported data is valuable for the future design and analysis of any protein fibrils binding to phase-separated lipid domains in model multi-component lipids membranes using either atomistic or coarse-grained molecular dynamics simulations. Additionally, this data can guide or validate future single-molecule experiments on fibril/membrane interactions in model or cell membranes.

Specifications table

**Table:** 

Subject	Modeling and simulations
Specific subject area	Analysis of lipid conformations and beta-amyloid fibril binding behaviors in phase-separated lipid raft domains, from molecular dynamics simulated data.
Type of data	Table
	Image
	Figure
	Movie
How data were acquired	Molecular dynamics simulations of NMR-derived beta-amyloid fibrils and modeled phase-separated lipid raft membranes, using Martini Coarse-Grained Force Fields [2], molecular dynamics, and GROMACS analysis programs [3].
Data format	Raw
Parameters for data collection	Simulations were performed under physiologically relevant conditions: 300 K, NPT ensemble, 0.1 M sodium chloride salt solution for 20 microseconds for each fibril/raft complex.
Description of data collection	After positioning fibrils of different sizes, in three different initial locations above the phase-separated lipid rafts of different lipid compositions, molecular dynamics simulations were performed. The final 0–20 microsecond trajectories and energy files were analyzed to create the data.
Data source location	Trinity University
	San Antonio
	Texas 78,212
	USA
Data accessibility	With the article
Related research article	Sara Y. Cheng, Yiyi Cao, Marzieh Rouzbehani, and Kwan H. Cheng in “Coarse-Grained MD Simulations reveal Diverse Membrane-Bound Conformational States of Beta-Amyloid Fibrils of Various Sizes on Cholesterol-Enriched Phase-Separated Lipid Rafts With or Without Glycolipid and Oxidized Cholesterol” Biophysical Chemistry in press (DOI: 10.1016/j.bpc.2020.106355) [1].

## Value of the data

• The data provides useful information about lipid conformations, amyloid-fibril binding behaviours, kinetics, and membrane-bound states, of beta-amyloid fibrils on phase-separated lipid rafts, which mimic the plasma membranes of neurons.
• The data will benefit computational and experimental molecular biophysicists interested in amyloid fibril interactions with lipid membranes. Additionally, molecular and cell biologists who are interested in the membrane-disruptive mechanisms of toxic fibrils in model membranes and live cells.
• The fibril's membrane-bound orientations, e.g., surface-bound or inserted, and locations in different lipid domains, e.g., glycolipid or cholesterol-enriched (Lo) or -depleted (Ld), or mixed Lod region, will provide insight to guide future experiments to understand the lipid composition and structures on toxic amyloid binding to cell membranes. The information is also useful for the future design of drug-interventions and new imaging markers, targeting membrane-bound states of amyloid fibrils.
• The simulated data will guide the design of proteins that bind to more complex lipid membranes, containing multiple lipid components and varying domain sizes and structures, that provide a more realistic representation of the complex cell membranes.

## 1 Data description

### 1.1 Structures of lipids and beta-amyloid fibrils

**Fig. 1:**
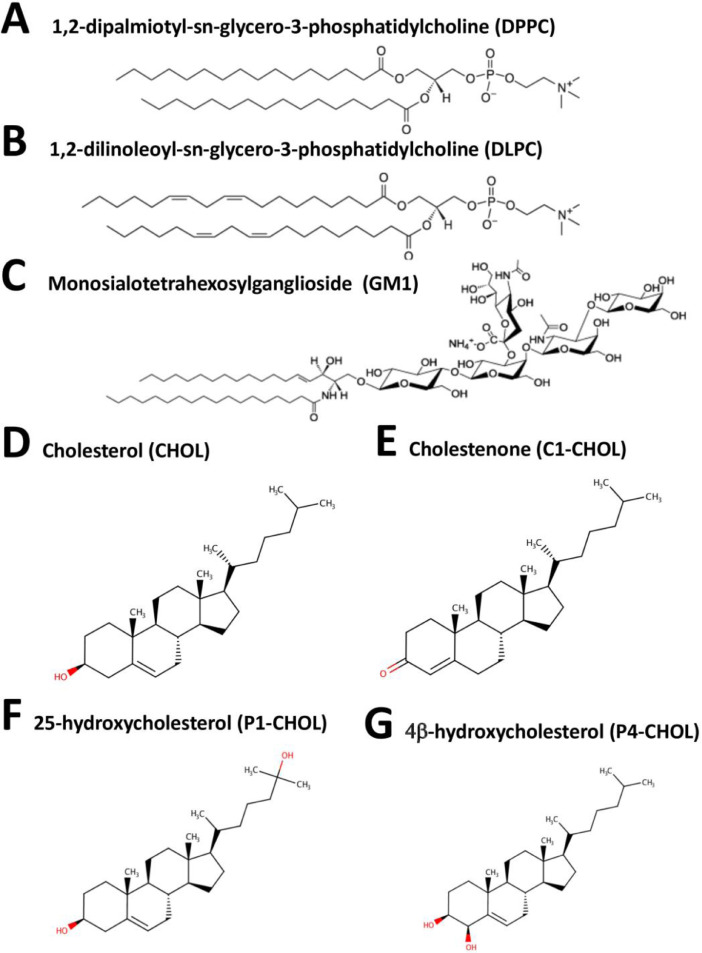
Chemical structure of DPPC (A), DLPC (B), GM1 (C), Cholesterol (D), Cholestenone (E), 25-hydroxycholesterol (F) and 4β-hydroxycholesterol (G). The corresponding sterols modeled in this coarse-grained work, i.e., CHOL, C1-CHOL, P1-CHOL, and P4-CHOL are shown.

Fig. 1 shows the chemical structures of saturated phospholipid, 1,2-dipalmitoyl-sn‑glycero-3-phosphatidylcholine (DPPC), unsaturated phospholipid, 1,2-dilinoleoyl-sn‑glycero-3-phosphatidylcholine (DLPC), glycolipid, monosialotetrahexosylganglioside (GM1), cholesterol (CHOL), and three oxidized cholesterols: cholestenone (C1-CHOL), 25-hydroxycholesterol (P1-CHOL) and 4b-hydroxycholesterol (P4-CHOL). The coarse-grained forms of these lipid molecules were used to constructed the multiple component lipid rafts. Chemical structure of each lipid can also be obtained from PubChem.

DPPC: https://pubchem.ncbi.nlm.nih.gov/compound/1_2-Dipalmitoylphosphatidylcholine

DLPC: https://pubchem.ncbi.nlm.nih.gov/compound/1_2-Dilinoleoyl-SN-glycero-3-phosphocholine

GM1: https://pubchem.ncbi.nlm.nih.gov/compound/Ganglioside-GM1

CHOL: https://pubchem.ncbi.nlm.nih.gov/compound/Cholesterol

C1-CHOL: https://pubchem.ncbi.nlm.nih.gov/compound/91477

P1-CHOL: https://pubchem.ncbi.nlm.nih.gov/compound/25-Hydroxycholesterol

P4-CHOL: https://pubchem.ncbi.nlm.nih.gov/compound/3247060

The chemical structure data files (in sdf format) are given in Supplementary Data (S01).

**Fig. 2:**
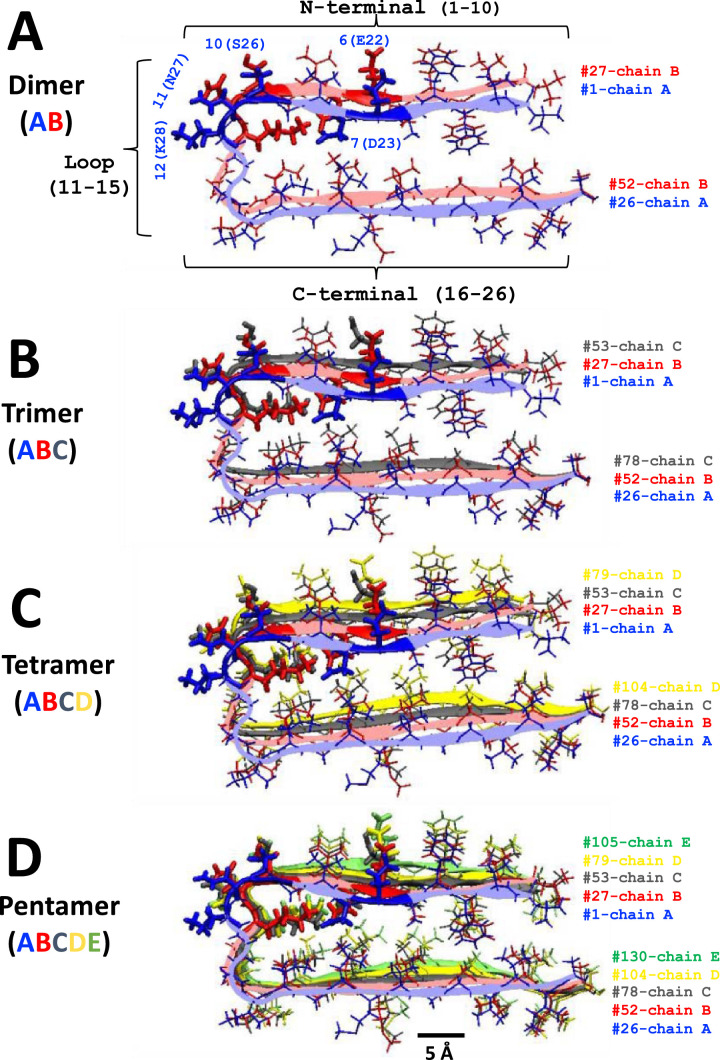
Structure of beta-amyloid fibrils (Aβ_17–42_)*_n_* of different sizes, dimer or *n* = 2 (A), trimer or *n* = 3 (B), tetramer or *n* = 4 (C) and pentamer or *n* = 5 (D). Residue numbers of individual fibril chains, 1–26 (chain A), 27–52 (chain B), 53–78 (chain C), 79–104 (chain D), and 105–130 (chain E) are labeled in blue, red, gray, yellow, and green, respectively. An identical color scheme is used to highlight the fibril backbone (ribbon and loop) and side chains (licorice). The polar residues 6 (E22), 7 (D23), 10 (S26), 11 (N27), and 12 (K28) are shown in thicker licorice. Based on the secondary structure, residues 1–10 (N-terminal), and residues 16–26 (C-terminal) consist of mainly beta-sheets, and the region in between, i.e., residues 11–15 (Loop), is mainly random. A scale bar of 5 Å is shown.

Fig. 2 shows the atomistic structures of beta-amyloid fibrils (PDB: 2BEG) obtained from the experimentally derived pentamer fibril NMR structure [4]. Smaller (dimer, trimer and tetramer) fibril structures were extracted from the original pentamer. All coarse-grained (CG) fibrils (dimer to pentamer) shown in the main article [1] were obtained by using a forward-mapping, or atomistic-to-CG, procedure [5]. The atomistic structure of fibrils can be downloaded from Protein Data Bank (PDB) (https://www.rcsb.org/structure/2BEG). The structure file of beta-amyloid fibril pentamer, 2beg.pdb, obtained from PDB is given in Supplementary Data (S02).

### 1.2 Structural characterization of lipids in phase-separated domains of lipid rafts

**Fig. 3:**
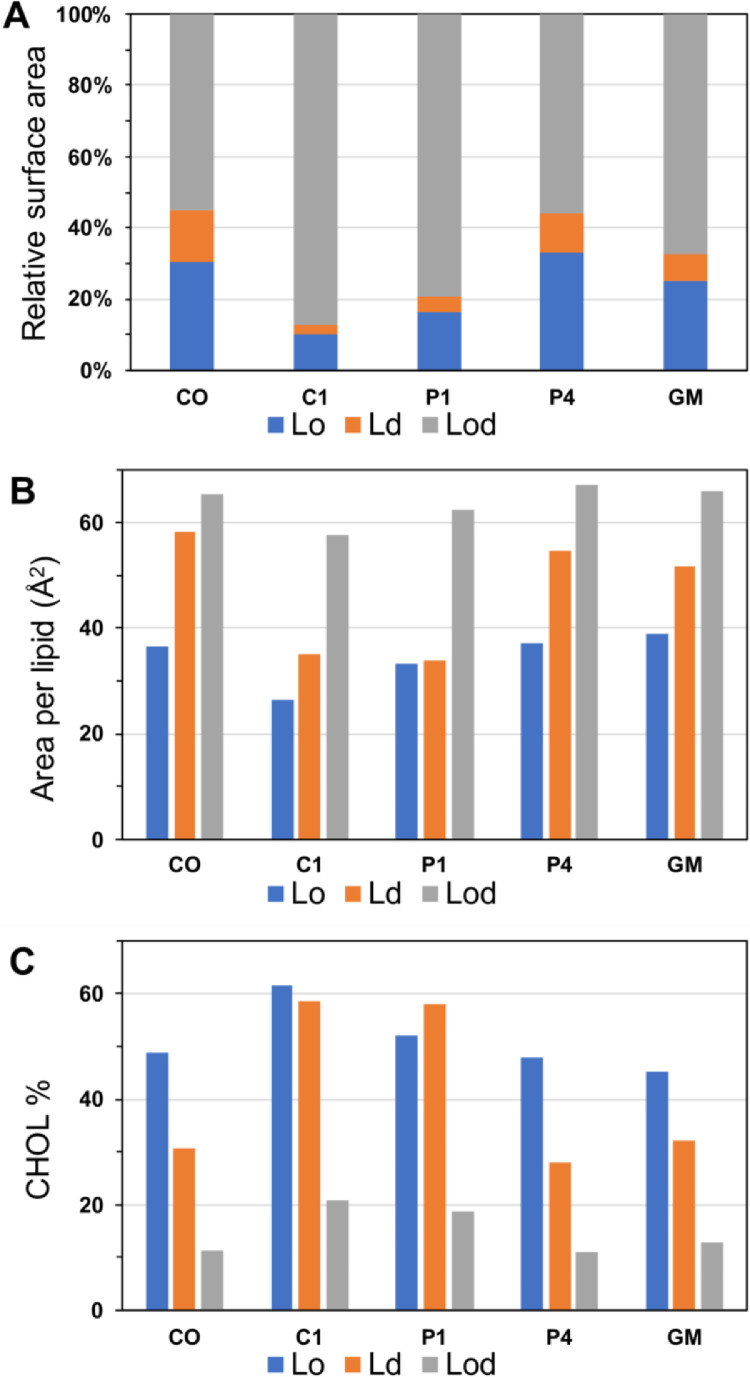
Relative surface area percentage (A), area per lipid (B) and cholesterol %, defined as number of CHOL/total number of lipids, (C) of each lipid domain (Lo, Ld or Lod) of CO-raft, C1-raft, P1-raft, P4-raft, and GM-raft.

Fig. 3 shows the relative surface area percentage of CHOL, area per lipid of CHOL, and CHOL% in each phase-separated liquid-ordered (Lo), liquid-disorder (Ld) or mixed Lo and Ld (Lod) domain in CO-raft (DPPC/DLPC/CHOL), C1-raft (DPPC/DLPC/C1-CHOL), P1-raft (DPPC/DLPC/P1-CHOL), P4-raft (DPPC/DLPC/P4-CHOL), and GM-raft (DPPC/DLPC/CHOL/GM1). The surface area of each lipid domain was determined using a grid-based membrane analysis program [6]. All data (in EXCEL) are given in Supplementary Data (S03).

**Fig. 4:**
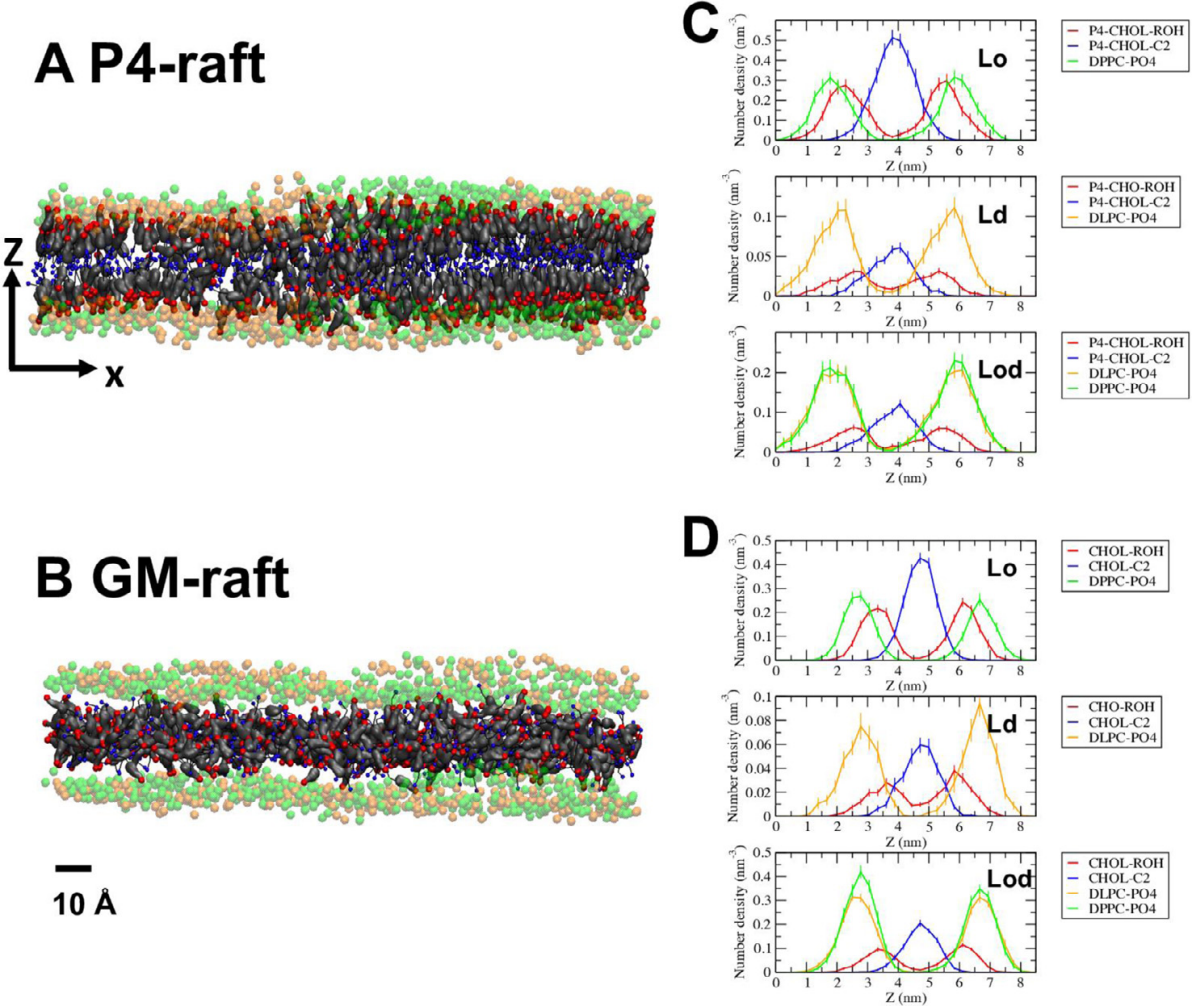
Representative transverse views of PO4 headgroups of DPPC in green, DLPC in orange, GM1 in purple, and ROH headgroups in red and C2 tail-groups in blue of CHOL or modified CHOL for P4-raft (A), and GM-raft (B). The time-averaged number density distribution of each lipid group in the Lo (upper panel), Ld (middle panel) and Lod (lower panel) over the last 5 µs of the 0 to 20 µs simulation for P4-raft (C), and GM-raft (D) were calculated from 50 volume slices, each with ∼ 0.25 nm thickness, along z, and covering the entire surface area in the x-y plane. The uncertainty, SE of mean, is also given. A scale bar of 10 Å is shown.

Fig. 4 shows the transverse views of lipid phosphate (PO4) headgroup and cholesterol headgroup and tail group in P4-raft and GM-raft, containing DPPC/DLPC/P4-CHOL, and DPPC/DLPC/CHOL/GM1. The number density vs. distance along the z-axis, or number density distributions, of those groups in each lipid domain, Lo, Ld, or Lod, of the two rafts are presented. The image files (in PNG format) and number distribution data (in agr format of xmgrace [7]) for P4-raft and GM-raft are given in Supplementary Data (S04).

**Fig. 5:**
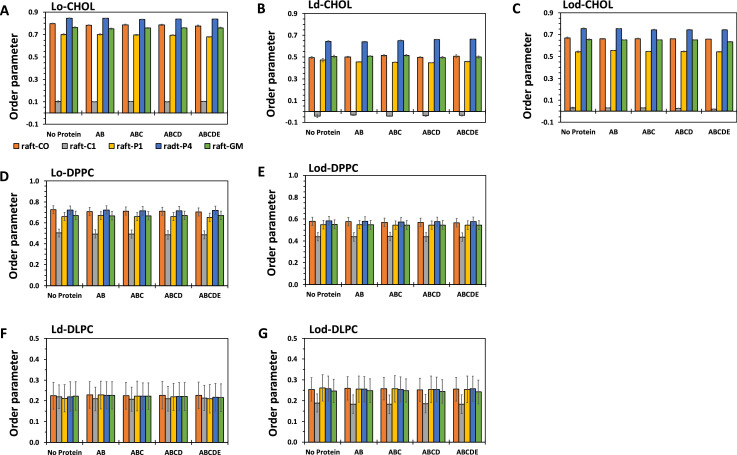
Time-averaged orientational order parameters of the ring group of CHOL (A)–(C) and acyl chains of DPPC (D) and (E) and DLPC (F) and (G) in Lo (A) and (D), Ld (B) and (F), and Lod (C), (E), and (G) domains over the last 5 µs of the 0 to 20 µs of the simulation in the absence (no protein) and presence of fibrils of different sizes. The average of three simulation replicates of different initial fibril positions from each fibril/raft complex is shown. The uncertainty, SE of mean, of each value is also given.

Fig. 5 shows the time-averaged lipid orientational order parameters of the ring group of CHOL and acyl chains of DPPC and DLPC in each phase separated lipid domains, Lo, Ld, or Lod, over the last 5 µs of the simulations of CO-raft, C1-raft, P1-raft, P4-raft, and GM-raft, in the absence (no protein) or presence of amyloid fibrils of various sizes (AB, ABC, ABCD, and ABCDE). All data (in EXCEL) are given in Supplementary Data (S05).

### 1.3 Fibril-raft binding kinetics

**Fig. 6:**
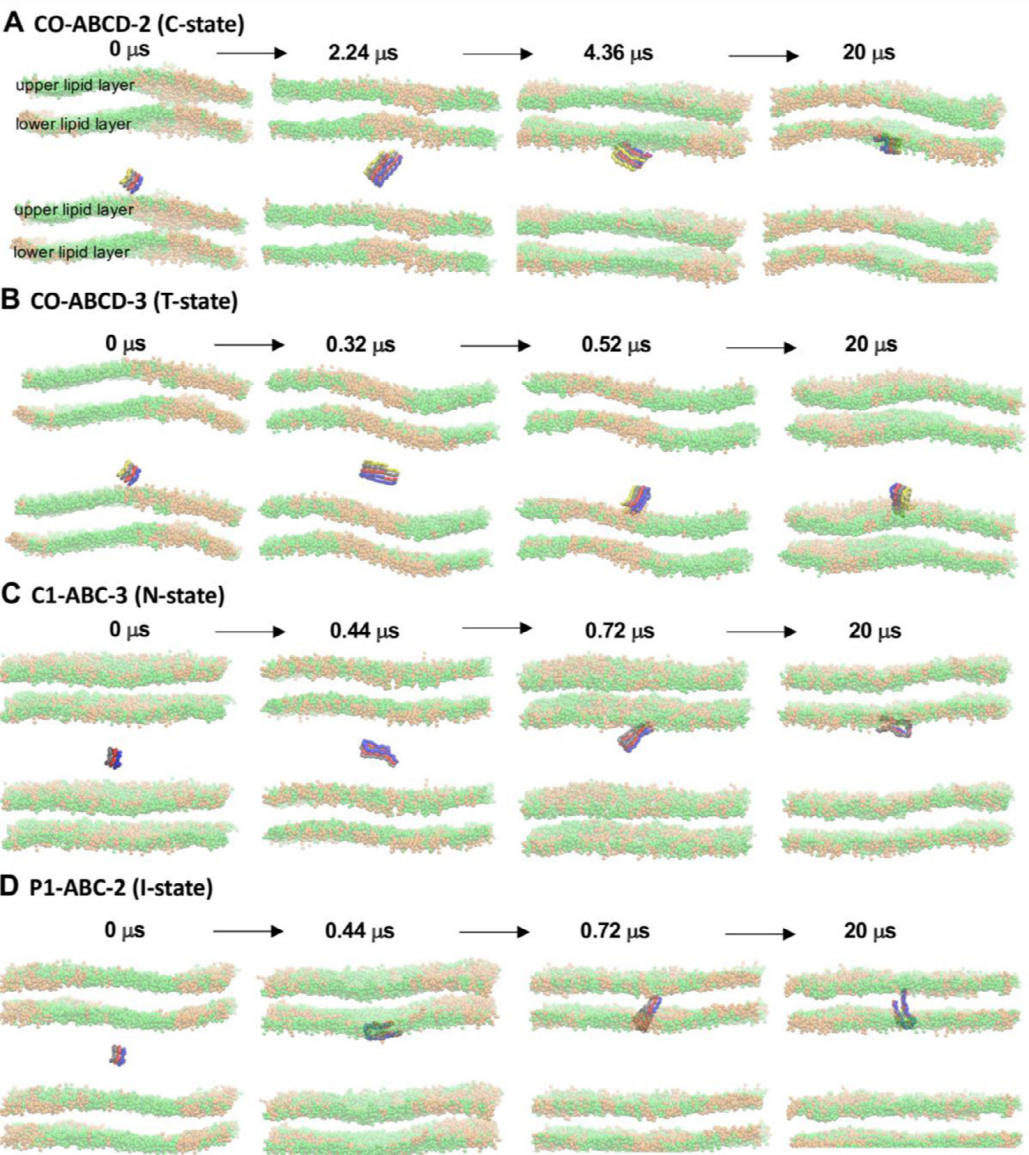
Transverse views of the orientations of fibrils (color ribbons) in solution and membrane-bound states at four selected times for CO-ABCD-2 in C-state (A), CO-ABCD-3 in T-state (B), C1-ABC-3 in C-state (C) and P1-ABC-2 in I state (D). A periodic image of the bilayer along z-axis is also presented. Headgroups of DPPC (green) and DLPC (orange) are shown. Upper lipid layer refers to the layer that the fibril was closer to at time zero. Lipid acyl chains and CHOL between the upper and lower layers are not shown for clarity.

Fig. 6 shows the snapshots of fibril binding and its final orientation in fibril/raft systems, CO-ABCD-2 in C-state, CO-ABCD-3 in T-state, C1-ABC-3 in C-state and P1-ABC-2 in I-state. The plot (in JPG) in given in Supplementary Data (S06-7)

**Fig. 7:**
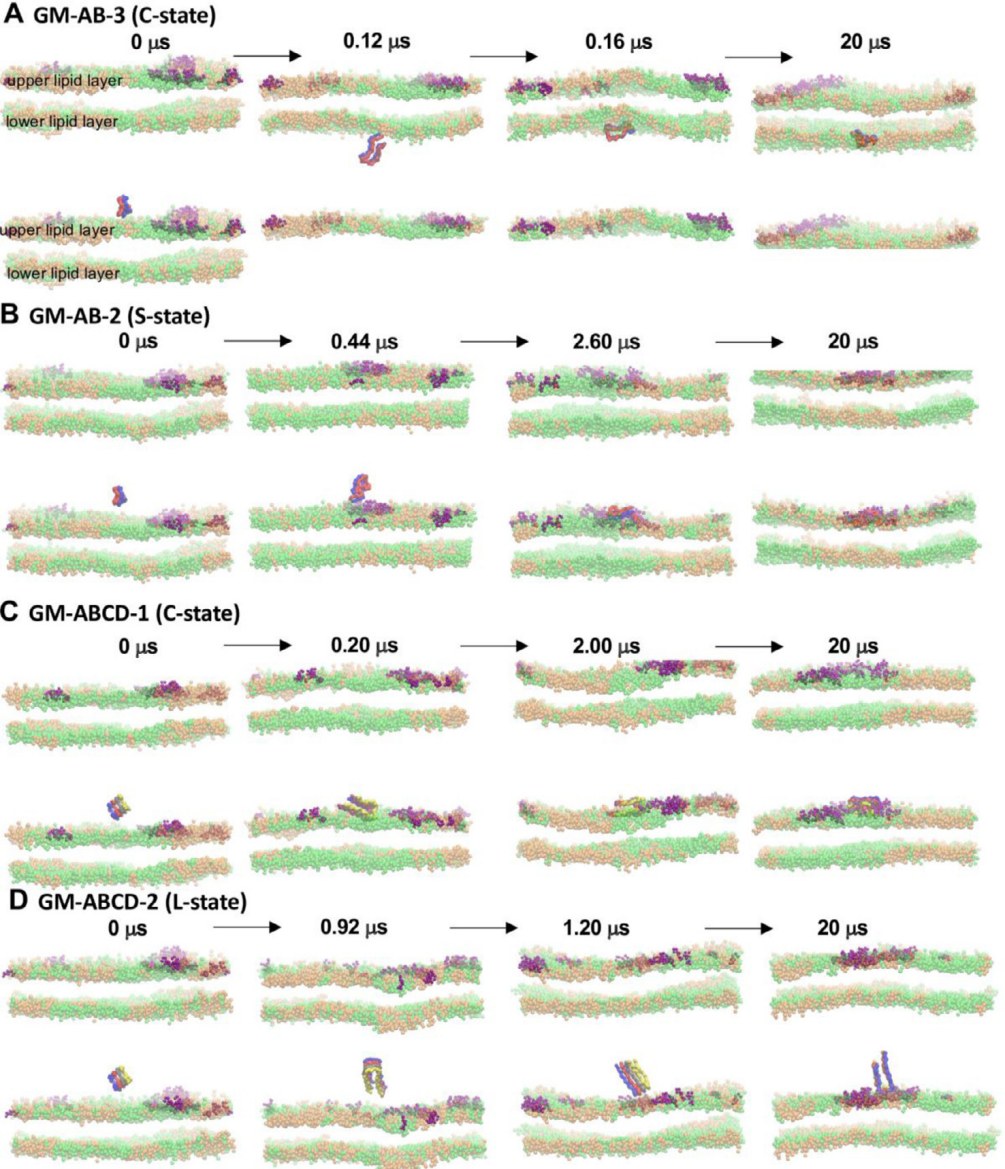
Transverse views of the orientations of fibrils (color ribbons) in solution and membrane-bound states at four selected times for GM-AB-3 in C-state (A), GM-AB-2 in S-state (B), GM-ABCD-1-in C-state (C) and GM-ABCD-2 in L state (D). See Fig. 6 legend for details.

Fig. 7 shows the snapshots of fibril binding and its final orientation in fibril/raft systems, GM-AB-3 in C-state, GM-AB-2 in S-state, GM-ABCD-1 (C-state) and GM-ABCD-2 (L-state). The plot (in JPG) in given in Supplementary Data (S06-7).

Table 1a, Table 1b, Table 1c show the binding time from fibril-lipid minimum atomic distance kinetics analysis [1] for each lipid, binding location (upper or lower lipid layer) and the equilibrated membrane-bound state of each simulation replicate. The data (in WORD) are given in Supplementary Data (Table S1(a)–(c))

**Table 1a:** Membrane binding times and membrane bound-orientations of fibrils in CO-raft and C1-raft.

Simulation replicate	Binding time (µs)			Binding conformation	
(0–20µs)	DPPC	CHOL	DLPC	Lipid layer	State
CO-AB-1	0.365	0.365	0.348	Lower	C
CO-AB-2	0.005	0.005	0.005		C
CO-AB-3	0.280	0.280	0.277		C
CO-ABC-1	0.265	0.265	0.253	Lower	C
CO-ABC-2	0.221	0.221	0.221	Lower	C
CO-ABC-3	1.330	1.330	1.325		C
CO-ABCD-1	0.889	0.889	1.088		C
CO-ABCD-2	4.210	4.270	4.210	Lower	C
CO-ABCD-3	0.439	0.451	0.430		**T**
CO-ABCDE-1	0.451	0.451	0.442		C
CO-ABCDE-2	0.348	0.348	0.348	Lower	C
CO-ABCDE-3	0.196	0.275	0.277	Lower	C
C1-AB-1	0.600	0.607	0.600		C
C1-AB-2	0.346	0.377	0.346		C
C1-AB-3	0.139	0.139	0.139		C
C1-ABC-1	0.140	0.160	0.140	Lower	**T**
C1-ABC-2	0.306	0.313	0.302		C
C1-ABC-3	0.690	0.700	0.660	Lower	**N**
C1-ABCD-1	0.331	0.355	0.321		C
C1-ABCD-2	0.856	0.856	0.842	Lower	C
C1-ABCD-3	0.168	0.196	0.168	Lower	**T**
C1-ABCDE-1	2.460	2.470	2.460	Lower	C
C1-ABCDE-2	0.340	0.342	0.340	Lower	C
C1-ABCDE-3	0.280	0.292	0.280	Lower	C

Fibril membrane binding times from fibril-lipid minimum distance kinetics of each simulation replicate is shown. Information about the fibril binding conformation, in terms of the location of fibril on lipid bilayer (upper or lower leaflet) and the membrane-bound state, for each simulation replicate is also presented. Starting from the solution phase, each fibril attached to either the upper or lower leaflet of the lipid bilayer after the 20 µs simulation, of each fibril/raft complex. For clarity, only the lower lipid layer binding events are identified below, if not indicated the fibril bound to the upper lipid layer. Membrane bound states other than C-states are indicated in bold.

**Table 1b:** Membrane binding times and membrane bound-orientations of fibrils in P1-raft and P4-raft.

Simulation replicate	Binding Time (µs)			Binding conformation	
(0–20µs)	DPPC	CHOL	DUPC	Lipid layer	State
P1-AB-1	0.237	0.237	0.237	Lower	C
P1-AB-2	0.422	0.422	0.418	Lower	C
P1-AB-3	0.317	0.317	0.317	Lower	C
P1-ABC-1	0.377	0.377	0.377	Lower	C
P1-ABC-2	0.331	0.334	0.333	Lower	**I**
P1-ABC-3	0.060	0.060	0.070	Lower	C
P1-ABCD-1	2.760	2.777	2.760		**N**
P1-ABCD-2	0.123	0.125	0.123	Lower	C
P1-ABCD-3	1.310	1.310	1.310	Lower	C
P1-ABCDE-1	0.629	0.629	0.629		C
P1-ABCDE-2	1.416	1.422	1.416	Lower	C
P1-ABCDE-3	0.640	0.640	0.640		C
P4-AB-1	2.020	2.000	1.900	Lower	C
P4-AB-2	0.145	0.145	0.192		C
P4-AB-3	0.421	0.421	0.416	Lower	C
P4-ABC-1	1.098	1.089	1.089	Lower	C
P4-ABC-2	1.062	1.065	1.052		C
P4-ABC-3	0.690	0.693	0.684		C
P4-ABCD-1	0.957	0.957	0.948	Lower	C
P4-ABCD-2	0.482	0.500	0.441		**T**
P4-ABCD-3	1.660	1.660	1.510		**T**
P4-ABCDE-1	0.075	0.075	0.075		C
P4-ABCDE-2	2.240	2.245	2.240	Lower	**T**
P4-ABCDE-3	3.270	3.270	3.270	Lower	**N**

Fibril membrane binding time from fibril-lipid minimum distance kinetics of each simulation replicate is shown. Also, information of the fibril binding conformation, in terms of the location of fibril on lipid bilayer (upper or lower leaflet) and the membrane-bound state, for each simulation replicate is presented. See footnote of Table 1a for description of table formatting.

**Table 1c:** Membrane binding times and bound-orientations of fibrils in fibril/GM-raft complexes.

Simulation replicate	Binding Time (µs)				Binding conformation	
(0–20µs)	DPPC	CHOL	DLPC	GM1	Lipid layer	State
GM-AB-1	0.626	0.679	0.626	0.377		**S**
GM-AB-2	2.296	2.296	2.296	0.244		**S**
GM-AB-3	0.059	0.059	0.059		Lower	C
GM-ABC-1	0.589	0.589	0.521	0.290		**N**
GM-ABC-2	0.936	0.948	0.936	5.840		**N**
GM-ABC-3	0.513	0.513	0.513	0.498		C
GM-ABCD-1	0.189	0.193	0.189	2.080		C
GM-ABCD-2				0.981		**L**
GM-ABCD-3	1.080	1.080	1.080	0.424		**N**
GM-ABCDE-1	0.805	0.812	0.805	0.612		C
GM-ABCDE-2	0.122	0.127	0.122		Lower	C
GM-ABCDE-3	9.120	9.140	9.120	0.388		**N**

Fibril membrane binding time from fibril-lipid minimum distance kinetics of each simulation replicate is shown. Also, information of the fibril binding conformation, in terms of the location of fibril on lipid bilayer (upper or lower leaflet) and the membrane-bound state, for each simulation replicate is presented. See footnote of Table 1a for description of table formatting.

**Movies**

Movies in animated GIF showing the binding kinetics of fibril of various sizes to lipid rafts from solution to membranes. For clarity, only the polar headgroup of DPPC, DLPC, and GM1 are shown. Each movie has a frame rate of 40 ns/frame for a total of 500 frames, spanning 0 to 20 µs of simulations.

CO-ABCD-2–0 to 20 µs-hgrs.gif - tetramer fibril (ABCD) with membrane-bound C-state on CO-raft.

CO-ABCD-3–0 to20 µs-hgrs.gif - tetramer fibril (ABCD) with membrane-bound T-state on CO-raft.

C1-ABC-3–0 to20 µs-hgrs.gif - trimer fibril (ABC) with membrane-bound N-state on C1-raft.

P1-ABC-2–0 to20 µs-hgrs.gif - trimer fibril (ABC) with membrane-bound I-state on P1-raft.

GM-ABCD-1–0 to 20 µs-hgrs.gif - tetramer fibril (ABCD) with membrane-bound C-state on GM-raft.

GM-ABCD-2–0 to 20 µs-hgrs.gif - tetramer fibril (ABCD) with membrane-bound l-state on GM-raft.

The movies can be viewed in Mendeley Data (DOI: 10.17632/ktrspy2swx.1) [8].

### 1.4 Fibril orientational order in lipid rafts

**Fig. 8:**
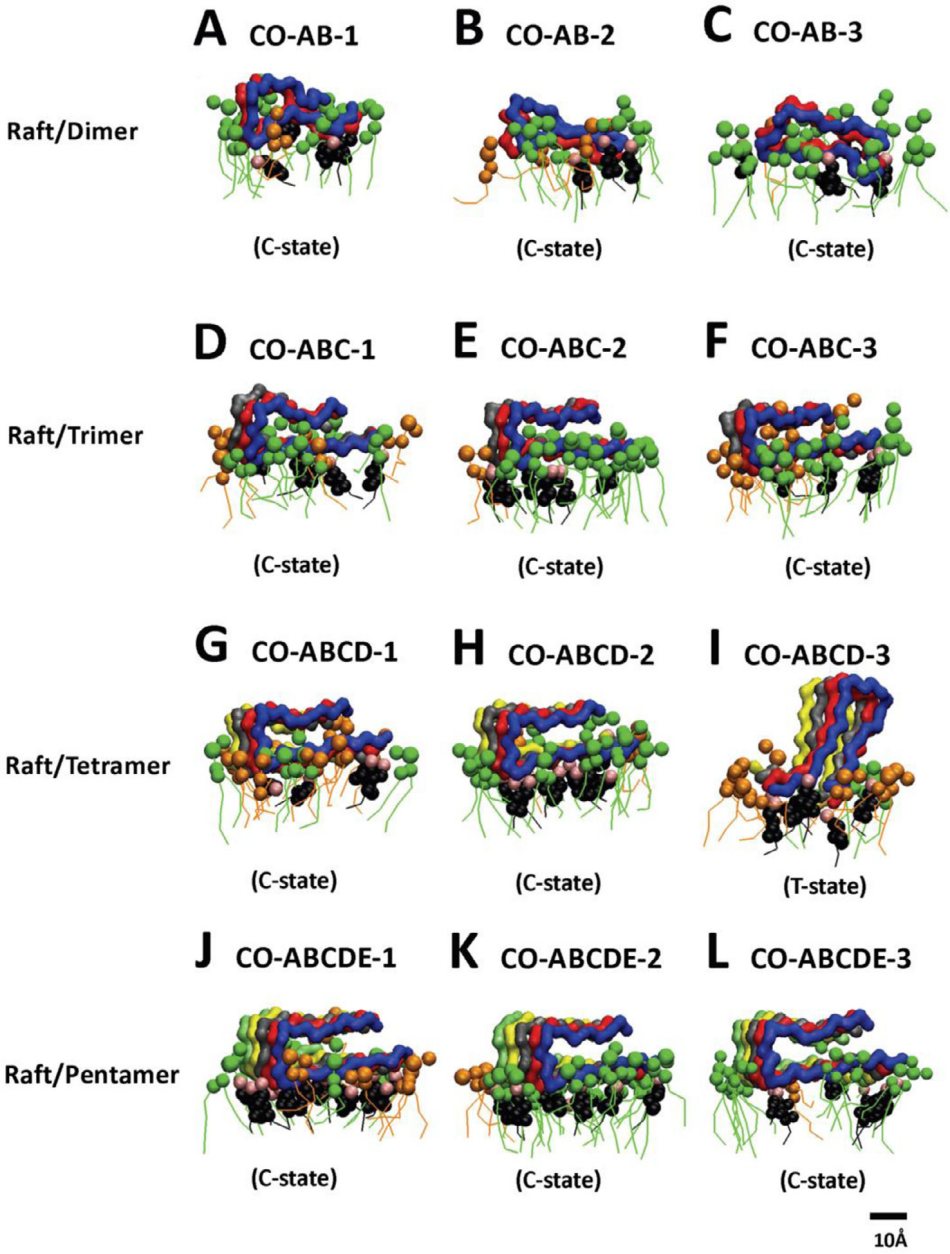
Membrane-bound states of fibrils of different sizes in fibril/CO-raft complexes. Transverse views of membrane-bound fibrils and their identified states after 20 µs simulations. DPPC in green, DLPC in orange, CHOL in black and the fibrils in color ribbons are shown.

**Fig. 9:**
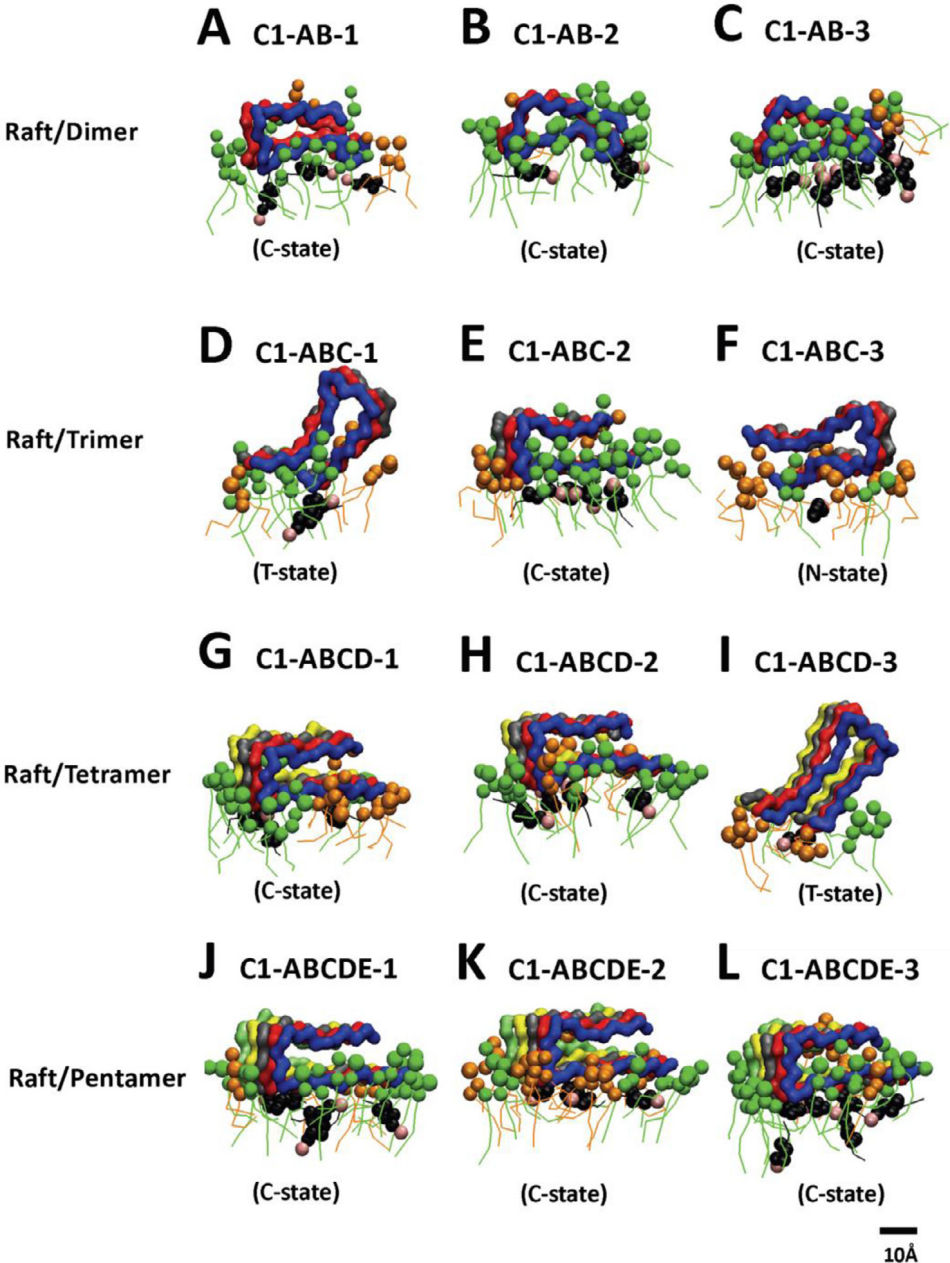
Membrane-bound states of fibrils of different sizes in fibril/C1-raft complexes. Transverse views of membrane-bound fibrils and their identified states after 20 µs simulations. DPPC in green, DLPC in orange, CHOL in black and fibril in color ribbons are shown.

**Fig. 10:**
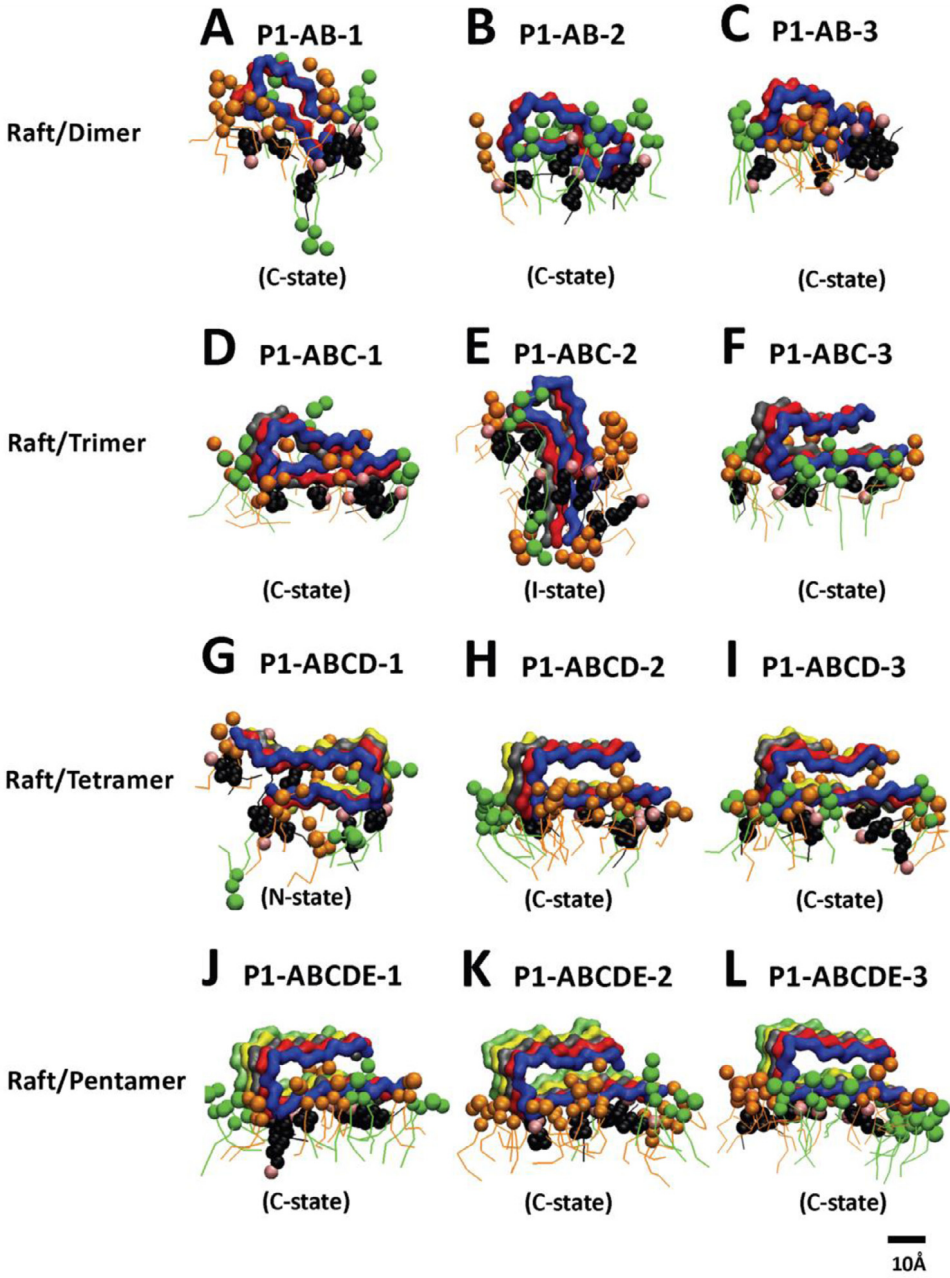
Membrane-bound states of fibrils of different sizes in fibril/P1-raft complexes. Transverse views of membrane-bounded fibrils and their identified states after 20 µs simulations. DPPC in green, DLPC in orange, CHOL in black and fibril in color ribbons are shown.

**Fig. 11:**
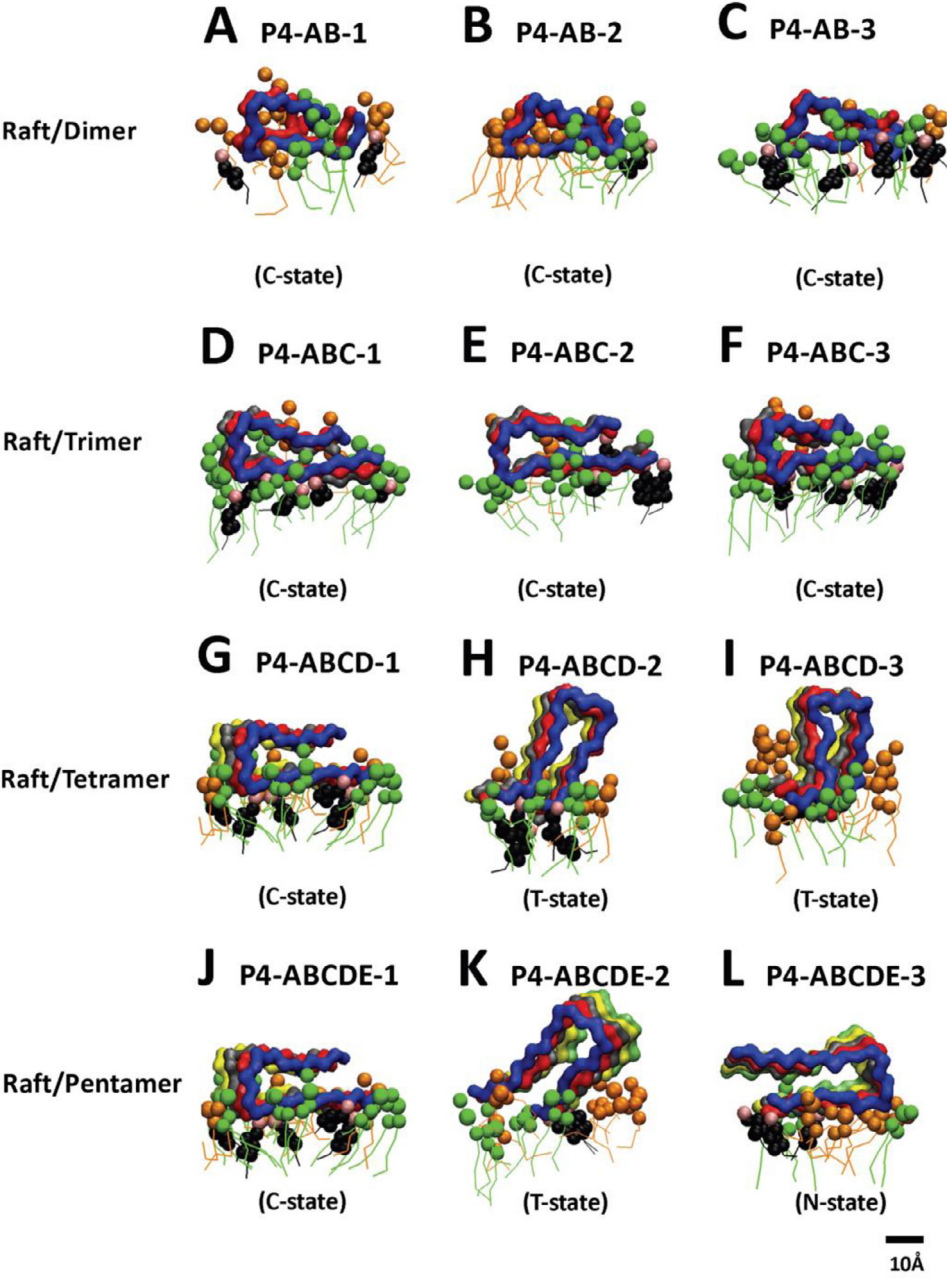
Membrane-bound states of fibrils of different sizes in fibril/P4-raft complexes. Transverse views of membrane-bound fibrils and their identified states after 20 µs simulations. DPPC in green, DLPC in orange, CHOL in black and fibril in color ribbons are shown.

**Fig. 12:**
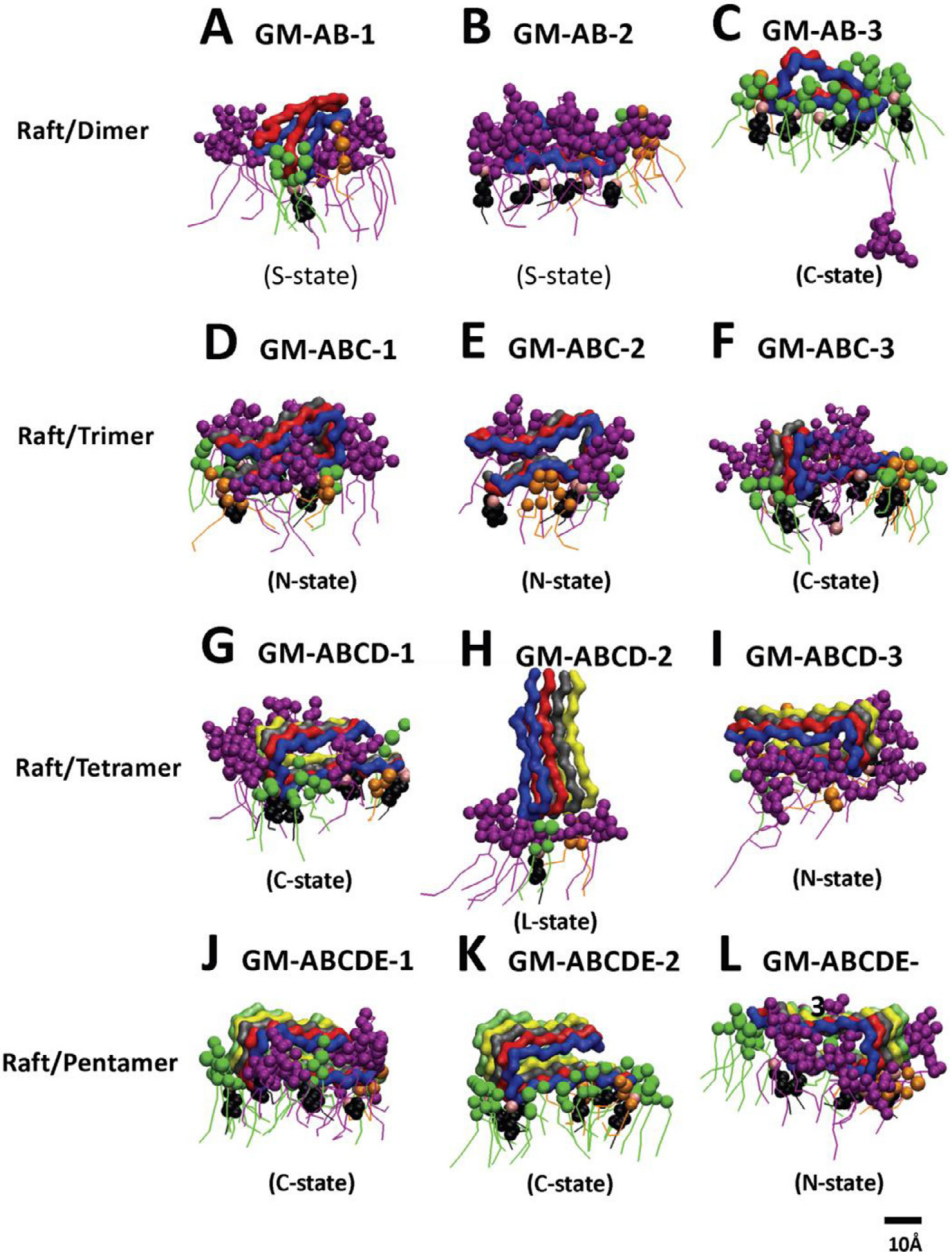
Membrane-bound states of fibrils of different sizes in fibril/GM-raft complexes. Transverse views of membrane-bound fibrils and their identified states after 20 µs simulations. DPPC in green, DLPC in orange, CHOL in black, GM1 in purple and fibril in color ribbons are shown.

Fig. 8, Fig. 9, Fig. 10, Fig. 11, Fig. 12 show the equilibrated membrane-bound states of fibrils at 2 µs for all 60 simulation replicates. The lipids within 0.5 nm from the membrane-bound fibril are shown. The structure files (in PDB) of all 60 replicates are given in Supplementary Data (S08-12).

**Fig. 13:**
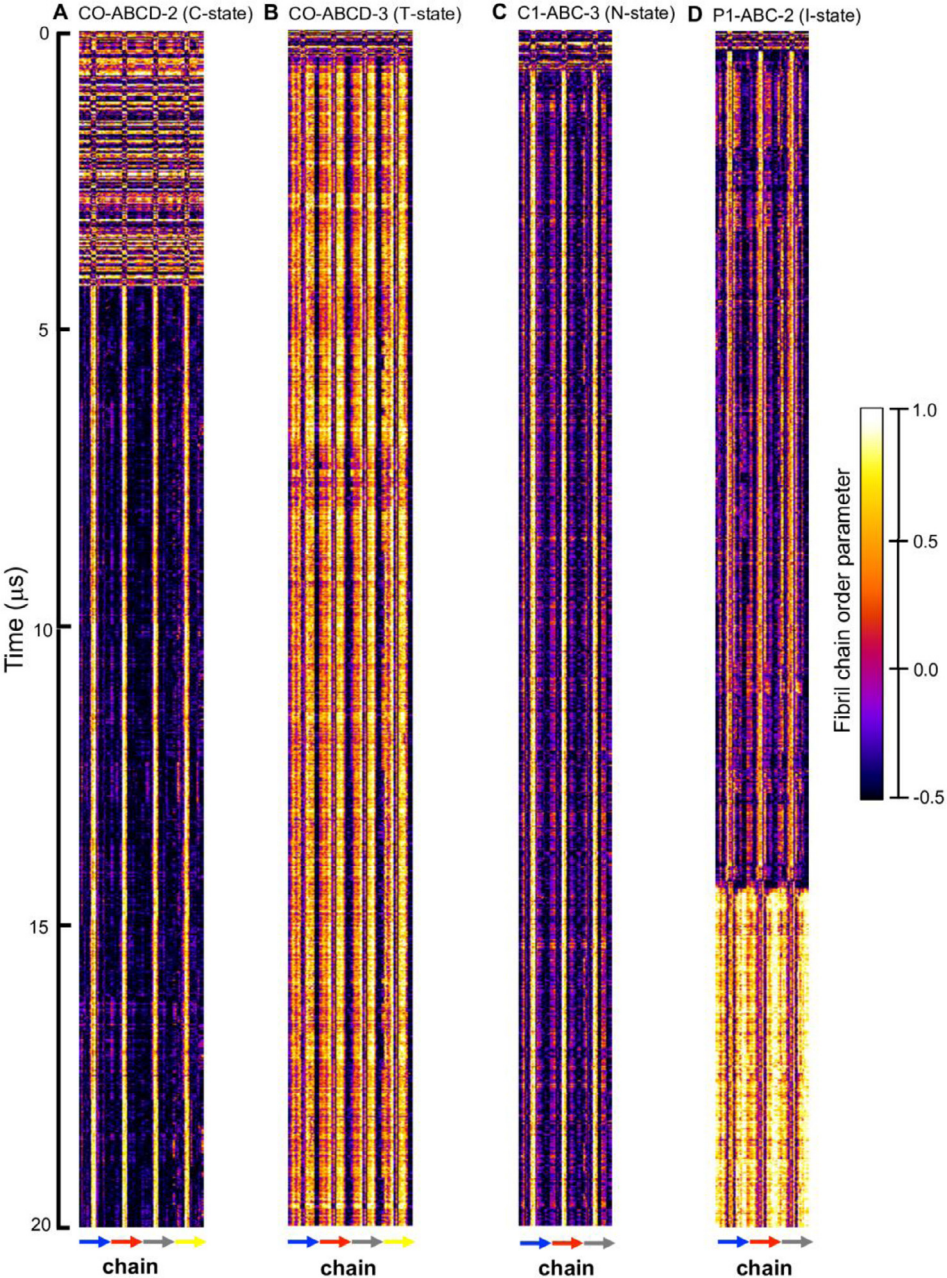
Fibril orientational order parameter vs. chain residue number (horizonal axis) vs. time (vertical axis) for simulation replicates, CO-ABCD-2 in C-state (A), CO-ABCD-3 in T-state (B), C1-ABC-3 in N-state (C), and P1-ABC-2 in I-state (D). The color bar (−0.5 to 1.0) for the order parameter is given. The chain residues from N- to C-terminus of chain A (blue), B (red), C (gray) and D (yellow) are given in color arrows.

**Fig. 14:**
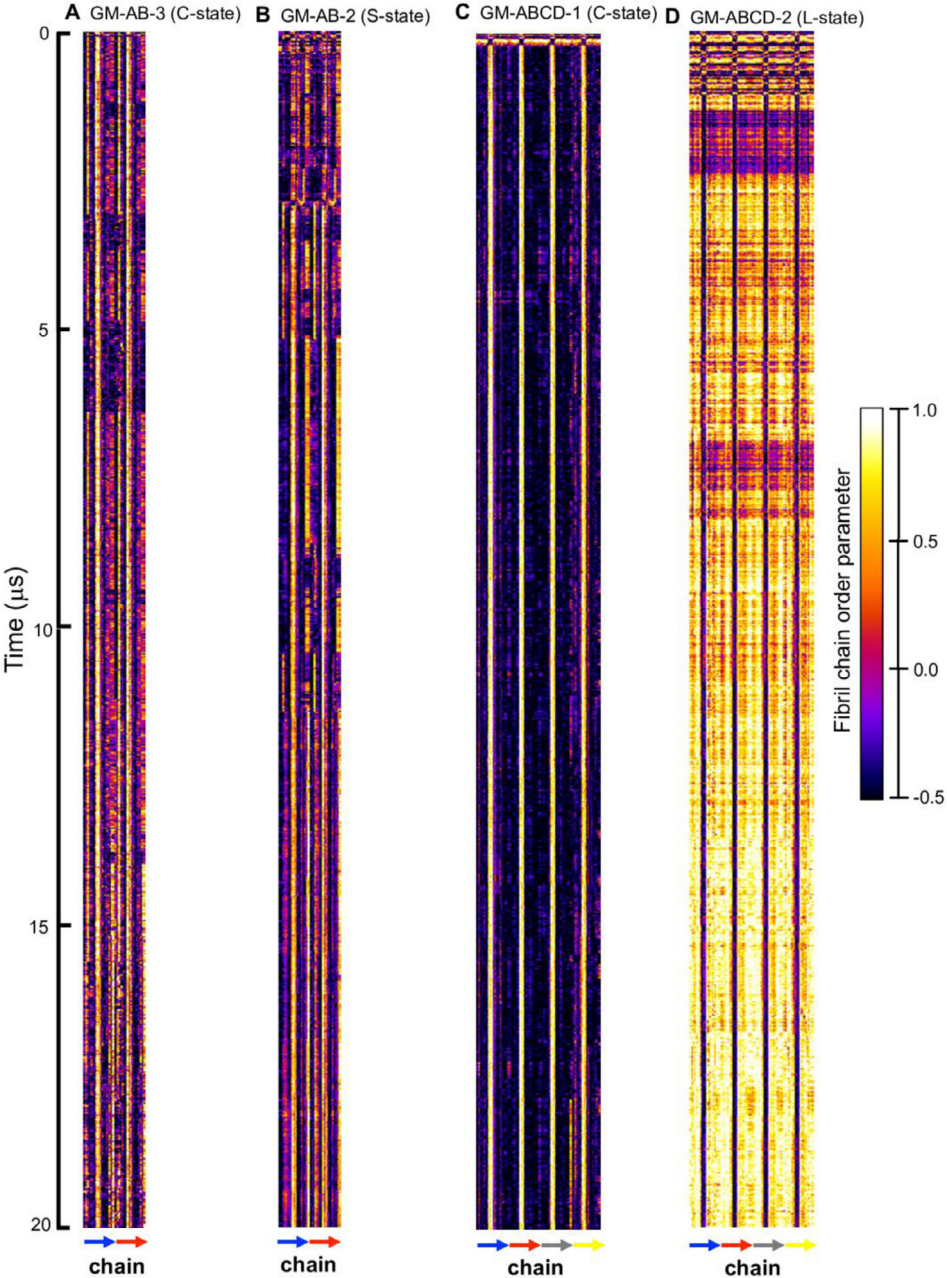
Fibril orientational order parameter vs. chain residue number (horizonal axis) vs. time (vertical axis) for simulation replicates, GM-AB-3 in C-state (A), GM-AB-2 in S-state (B), GM-ABCD-1 in C-state (C), and GM-ABCD-2 in L-state (D). The color bar (−0.5 to 1.0) for the order parameter is given. The chain residues from N- to C-terminus of chain A (blue), B (red), C (gray) and D (yellow) are given in color arrows.

Figs. 13 and 14 show the fibril orientational order parameter (−0.5 to 1 with color bar) vs. residue location (horizontal or x-axis and in horizonal color arrows highlighting the fibril chains from N-terminus to C-terminus) and time (vertical or y-axis from 0 to 20 µs) for simulation replicates, CO-ABCD-2 in C-state, CO-ABCD-3 in T-state, C1-ABC-3 in N-state, P1-ABC-2 in I-state, GM-AB-3 in C-state, GM-AB-2 in S-state, GM-ABCD-1 in C-state and GM-ABCD-2 in l-state. The fibril orientational order was calculated from the *g_order* tool of GROMACS [3]. The color maps are given in Supplementary Data (S13-14).

**Fig. 15:**
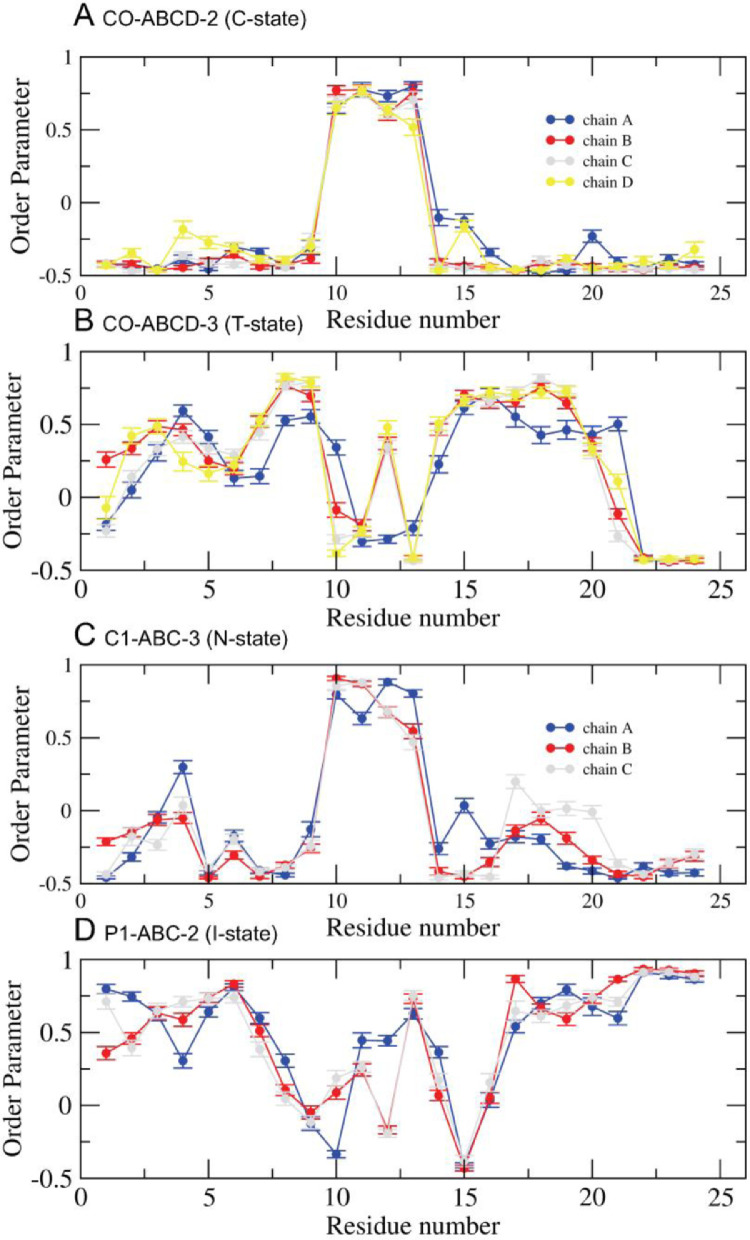
Time-averaged fibril orientational order parameter vs fibril residue number over the last 5 µs of the 0 to 20 µs simulation for CO-ABCD-2 in C-state (A), CO-ABCD-3 in T-state (B), C1-ABC-3 in N-state (C) and P1-ABC-2 in I-state (D) for chain A (blue), B (red), C (gray) and D (yellow). The uncertainty, SE of means, is shown.

**Fig. 16:**
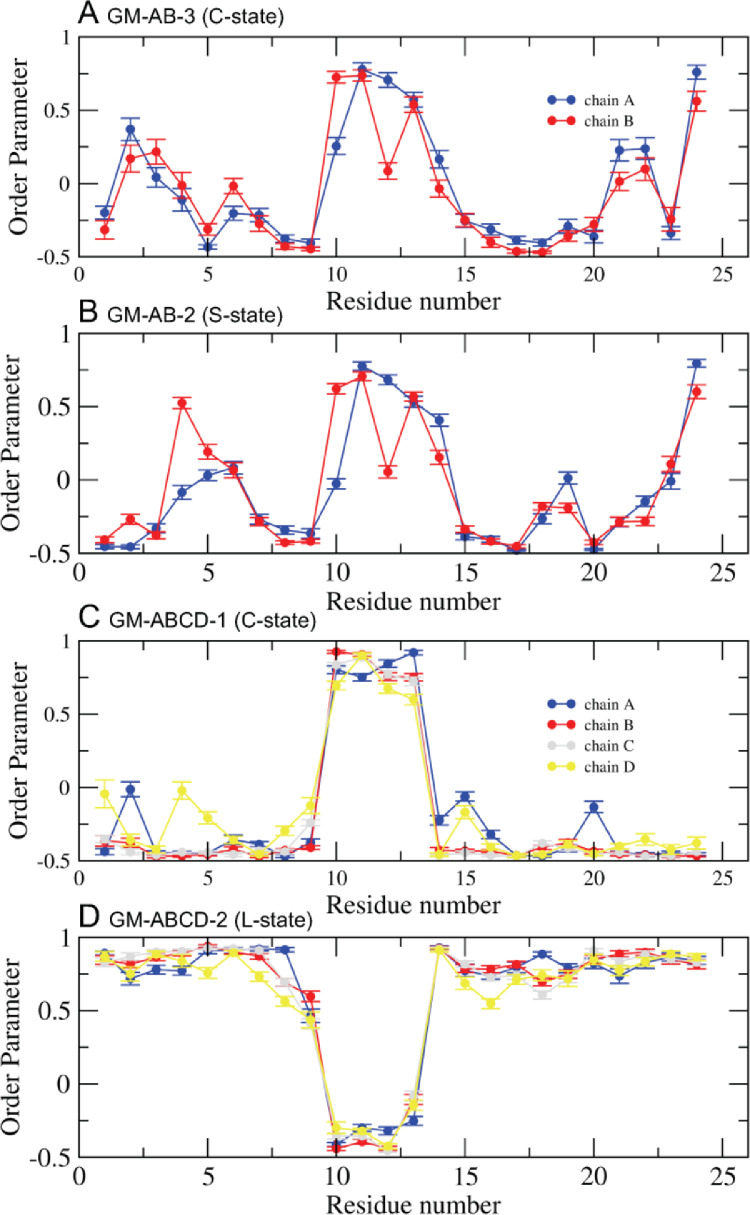
Time-averaged fibril orientational order parameter vs fibril residue number over the last 5 µs of the 0 to 20 ms simulation for GM-AB-3 in C-state (A), GM-AB-2 in S-state (B), GM-ABCD-1 in C-state (C) and GM-ABCD-2 in L-state (D) for chain A (blue), B (red), C (gray) and D (yellow). The uncertainty, SE of means, is shown.

Figs. 15 and 16 show the time-averaged fibril orientational parameter vs. residue number of the fibril over the last 5µs for simulation replicates, CO-ABCD-2 in C-state, CO-ABCD-3 in T-state, C1-ABC-3 in N-state, P1-ABC-2 in I-state, GM-AB-3 in C-state, GM-AB-2 in S-state, GM-ABCD-1 in C-state and GM-ABCD-2 in L-state. The plots (in agr of xmgrace [7]) are given in Supplementary Data (S15–16).

### 1.5 Minimum-distance analysis of fibril-lipid interactions

**Fig. 17:**
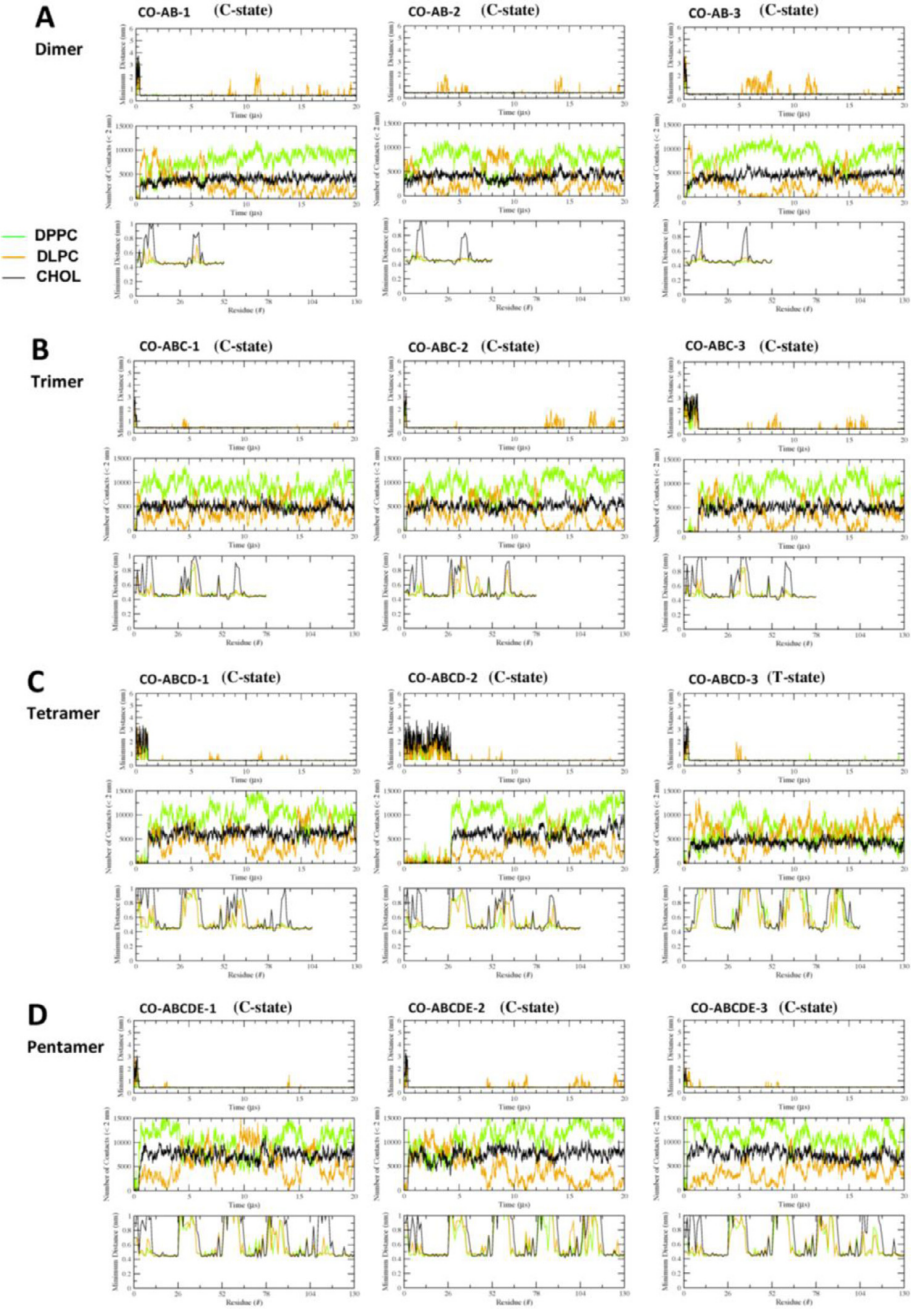
The minimum distance between the lipid and protein atoms vs. time (upper panel), number of contacts between lipid and protein atoms that are less than 2 nm vs. time (mid panel), and the minimum distance averaged over the last 5 µs vs. residue location of the fibril (lower panel) for CO-raft. The membrane-bound states are shown.

**Fig. 18:**
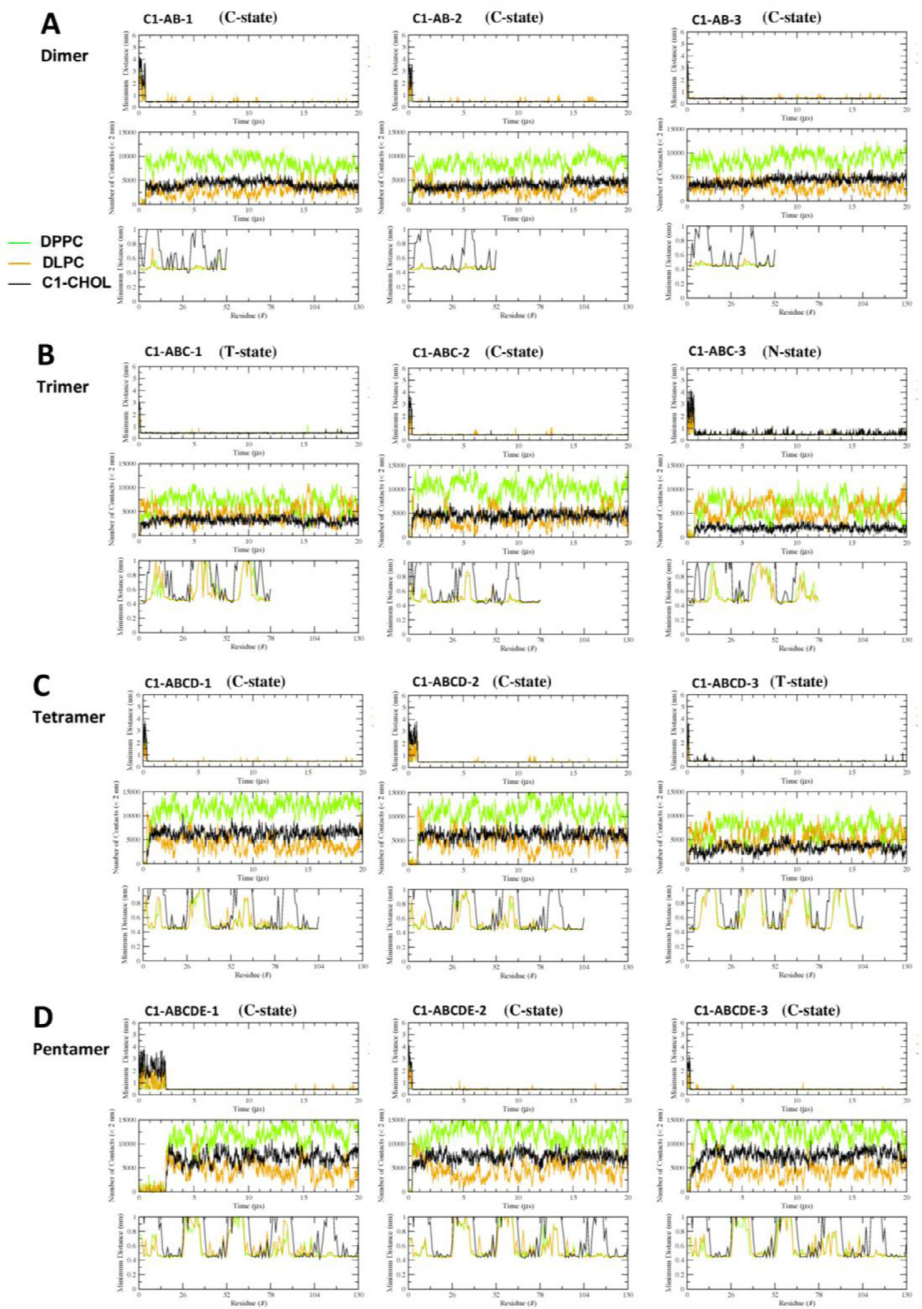
The minimum distance between the lipid and protein atoms vs. time (upper panel), number of contacts between lipid and protein atoms that are less than 2 nm vs. time (mid panel), and the minimum distance averaged over the last 5 µs vs. residue location of the fibril (lower panel) for C1-raft. The membrane-bound states are shown.

**Fig. 19:**
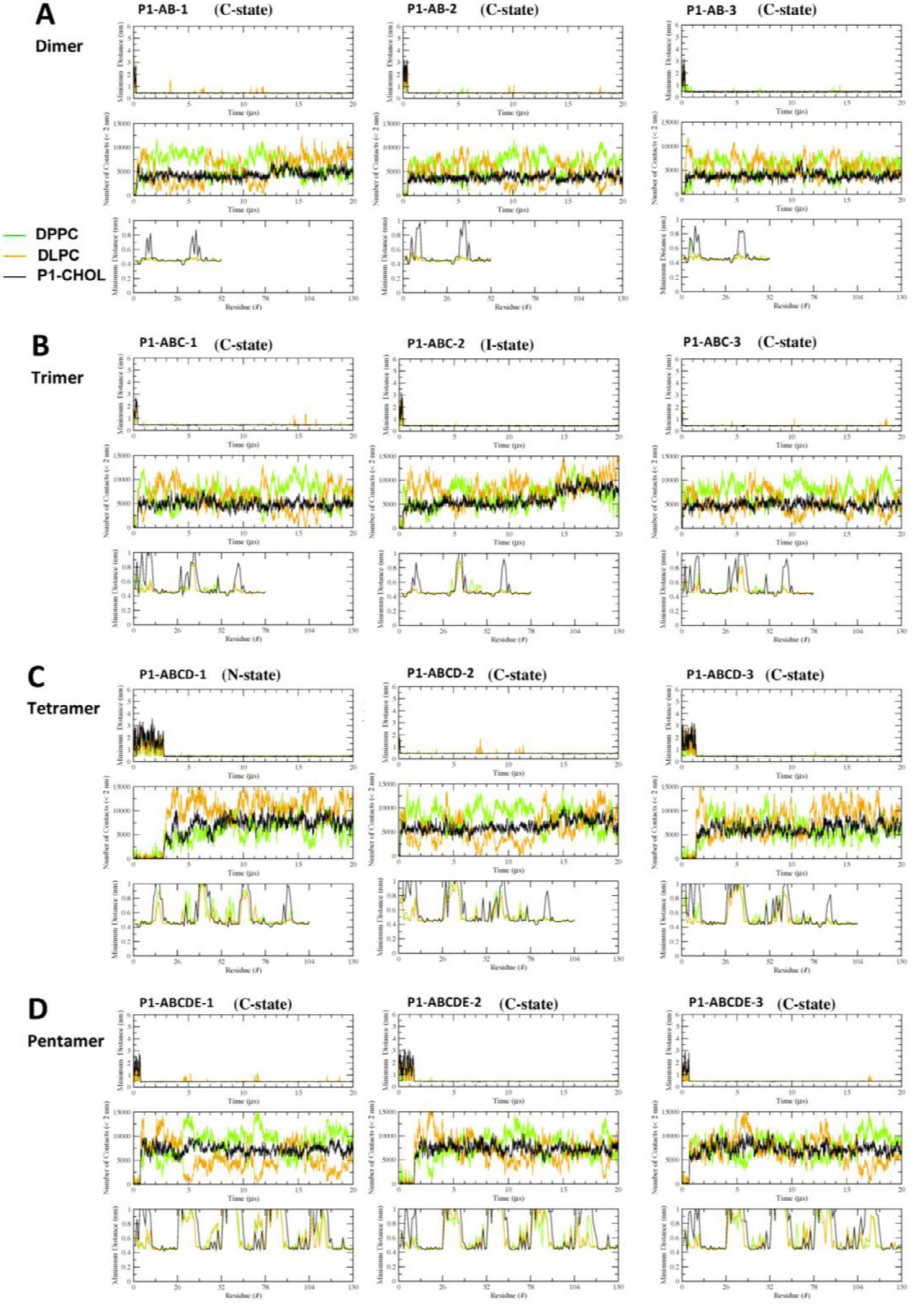
The minimum distance between the lipid and protein atoms vs. time (upper panel), number of contacts between lipid and protein atoms that are less than 2 nm vs. time (mid panel), and the minimum distance averaged over the last 5 µs vs. residue location of the fibril (lower panel) for P1-raft. The membrane-bound states are shown.

**Fig. 20:**
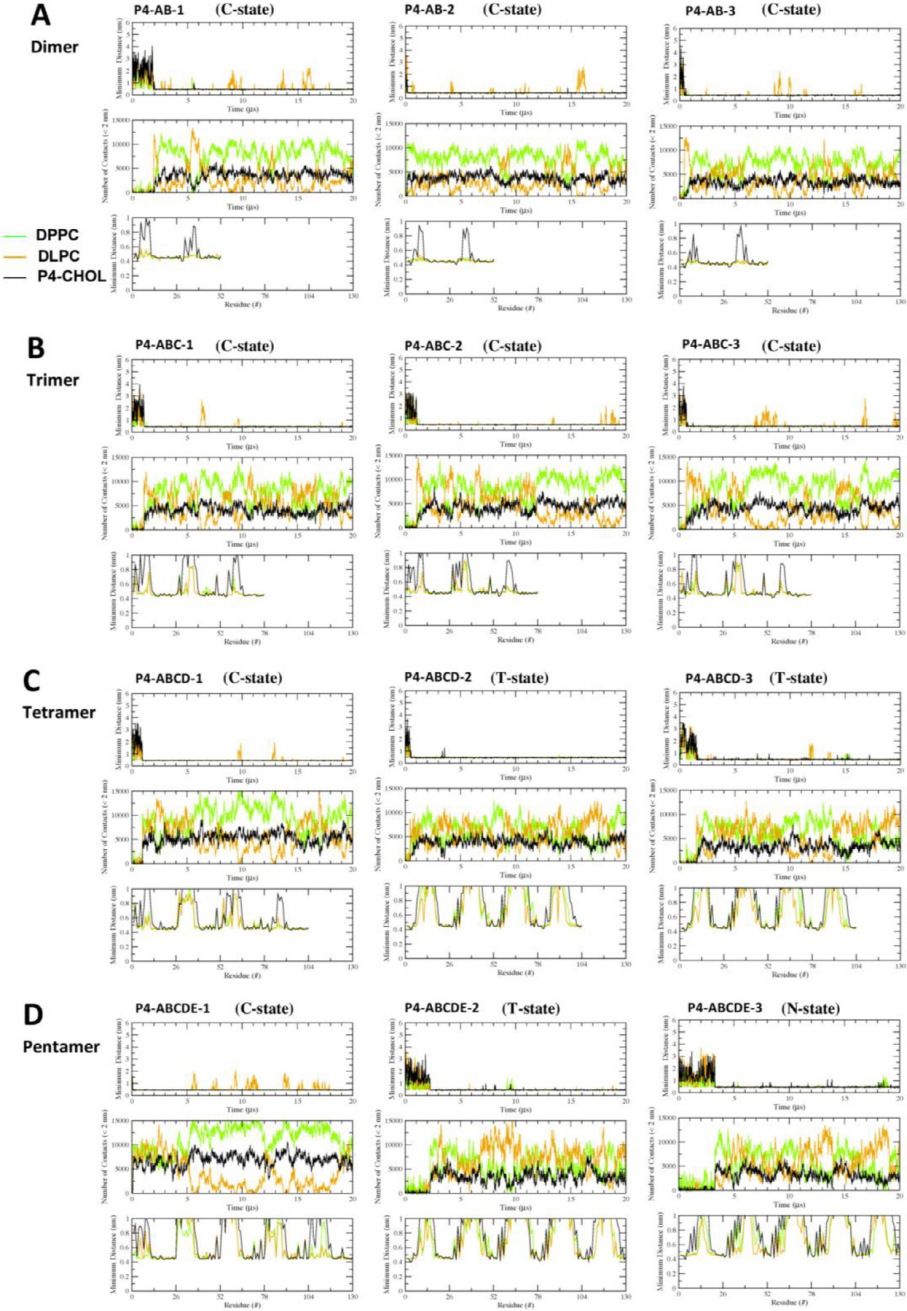
The minimum distance between the lipid and protein atoms vs. time (upper panel), number of contacts between lipid and protein atoms that are less than 2 nm vs. time (mid panel), and the minimum distance averaged over the last 5 µs vs. residue location of the fibril (lower panel) for P4-raft. The membrane-bound states are shown.

**Fig. 21:**
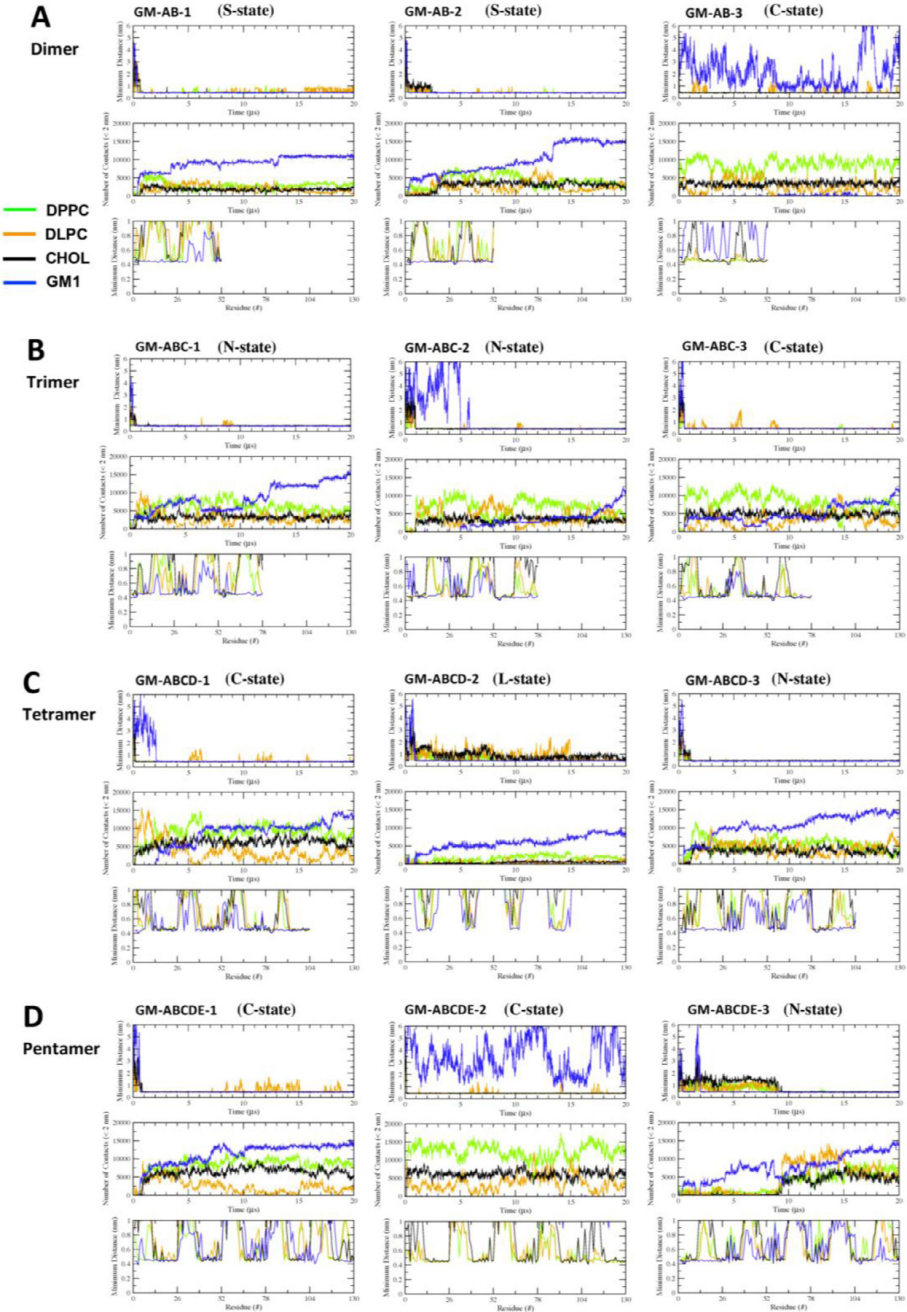
The minimum distance between the lipid and protein atoms vs. time (upper panel), number of contacts between lipid and protein atoms that are less than 2 nm vs. time (mid panel), and the minimum distance averaged over the last 5 µs vs. residue location of the fibril (lower panel) for GM-raft. The membrane-bound states are shown.

Fig. 17, Fig. 18, Fig. 19, Fig. 20, Fig. 21 show the minimum-distance analysis of all 60 simulation replicates. Each plot shows the minimum distance between atoms of fibril and lipid molecules vs. time (upper panel), number of contacts between lipid and fibril atoms (middle panel) within 2 nm vs. time, and time-averaged minimum distance between atoms of fibril and lipid molecules (lower panel) over the last 5 µs of simulations vs. fibril residue number. Plots of minimum-distance analysis (in agr format of xmgrace [7]) are given in Supplementary Data (S17-21).

**Fig. 22:**
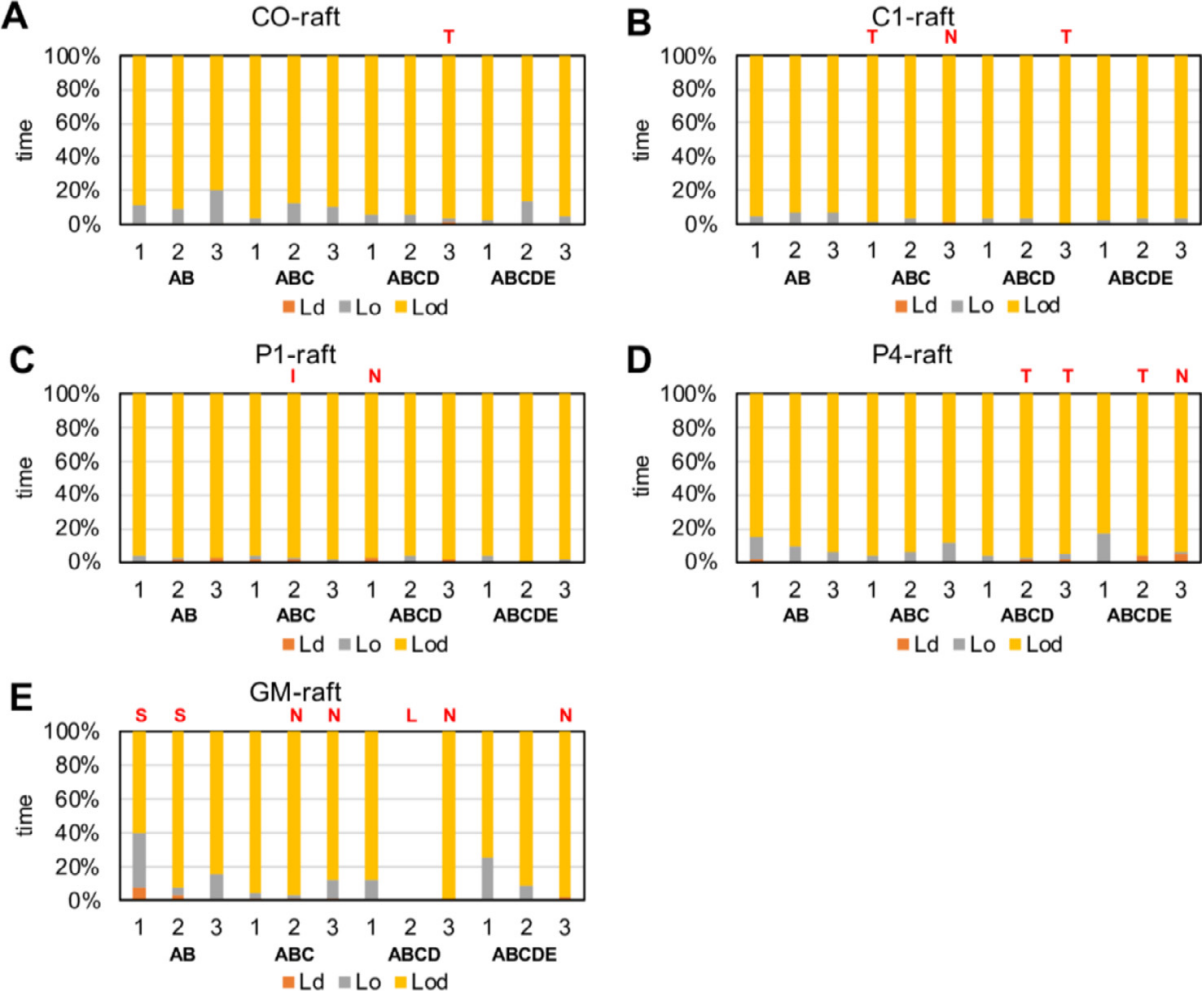
Domain residence time % of fibrils in Ld, Lo or Lod domain for each simulation replicate in CO-raft (A), C1-raft (B), P1-raft (C), P4-raft (D) and GM-raft (E). The membrane-bound state of each simulation replicate other than C-state is given.

Fig. 22 shows the domain resident time% of fibrils in Ld, Lo or Lod domain for all 60 simulation replicates. The Lo resident time is determined as the fraction of time in which the minimum distance between the atoms of fibril and DLPC is greater than 0.5 nm over the entire time of fibril contact with membrane. The Ld resident time is determined as the fraction of time in which the minimum distance between the atoms of fibril and DPPC is greater than 0.5 nm over the entire time of fibril contact with membrane. Finally, the Lod resident time is the time in which the minimum distance between the atoms of fibril and all DPPC and DLPC is less than 0.5 nm. The minimum fibril-lipid distance vs. time data in each lipid domain data for all replicates are given in the upper panel of the plots in Fig. 17, Fig. 18, Fig. 19, Fig. 20, Fig. 21. The analyzed data (in EXCEL) are given in Supplementary Data (S22).

**Fig. 23:**
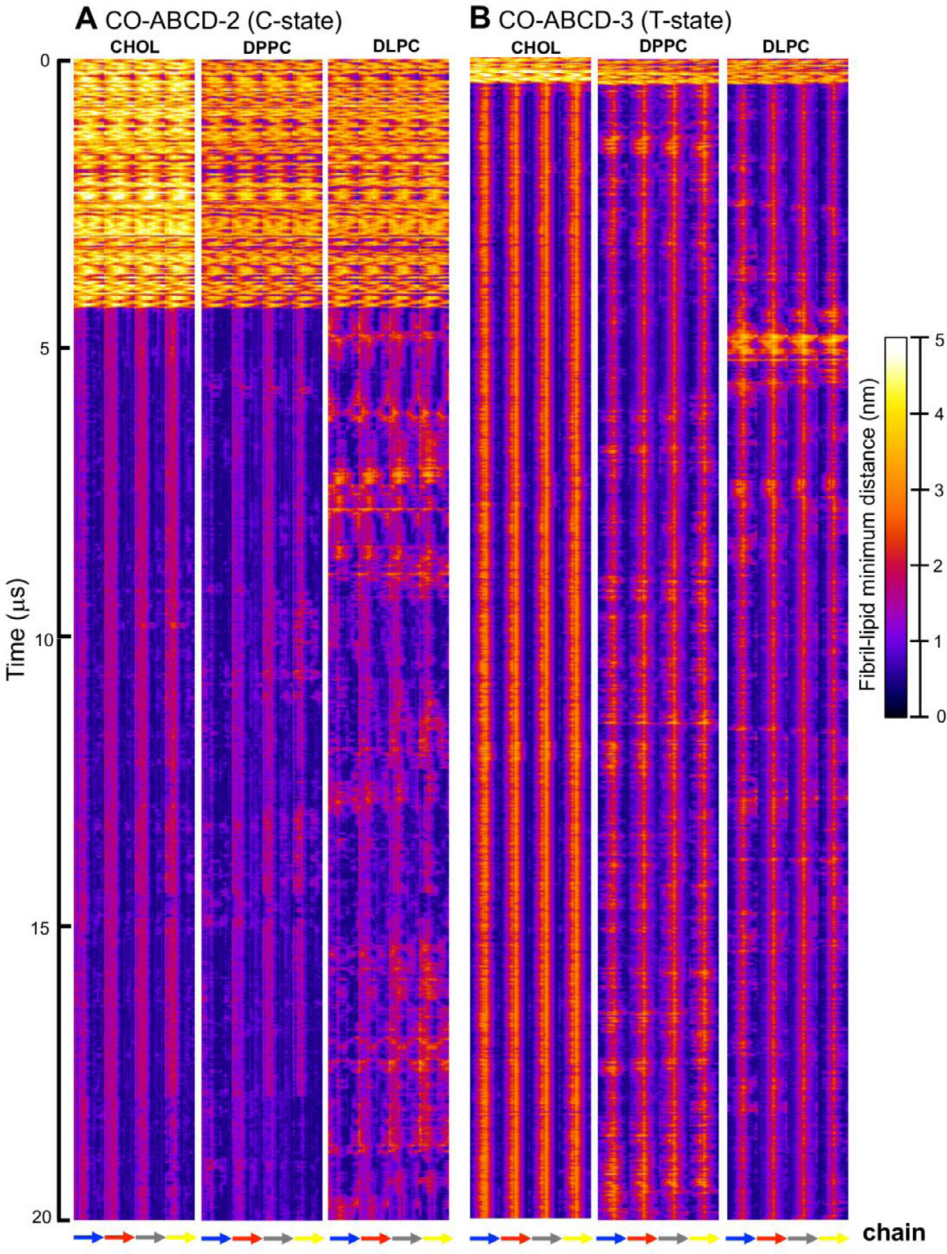
Fibril residue-lipid contact maps for CO-ABCD-2 in C-state (A) and CO-ABCD-3 in T-state (B) for CHOL, DPPC and DLPC. The chain residue (*x*-axis) and time (*y*-axis) are given. The color bar (0 to 5 nm) is also shown. The chain residues from N- to C-terminus of chains A (blue), B (red), C (gray) and D (yellow) are given in color arrows.

**Fig. 24:**
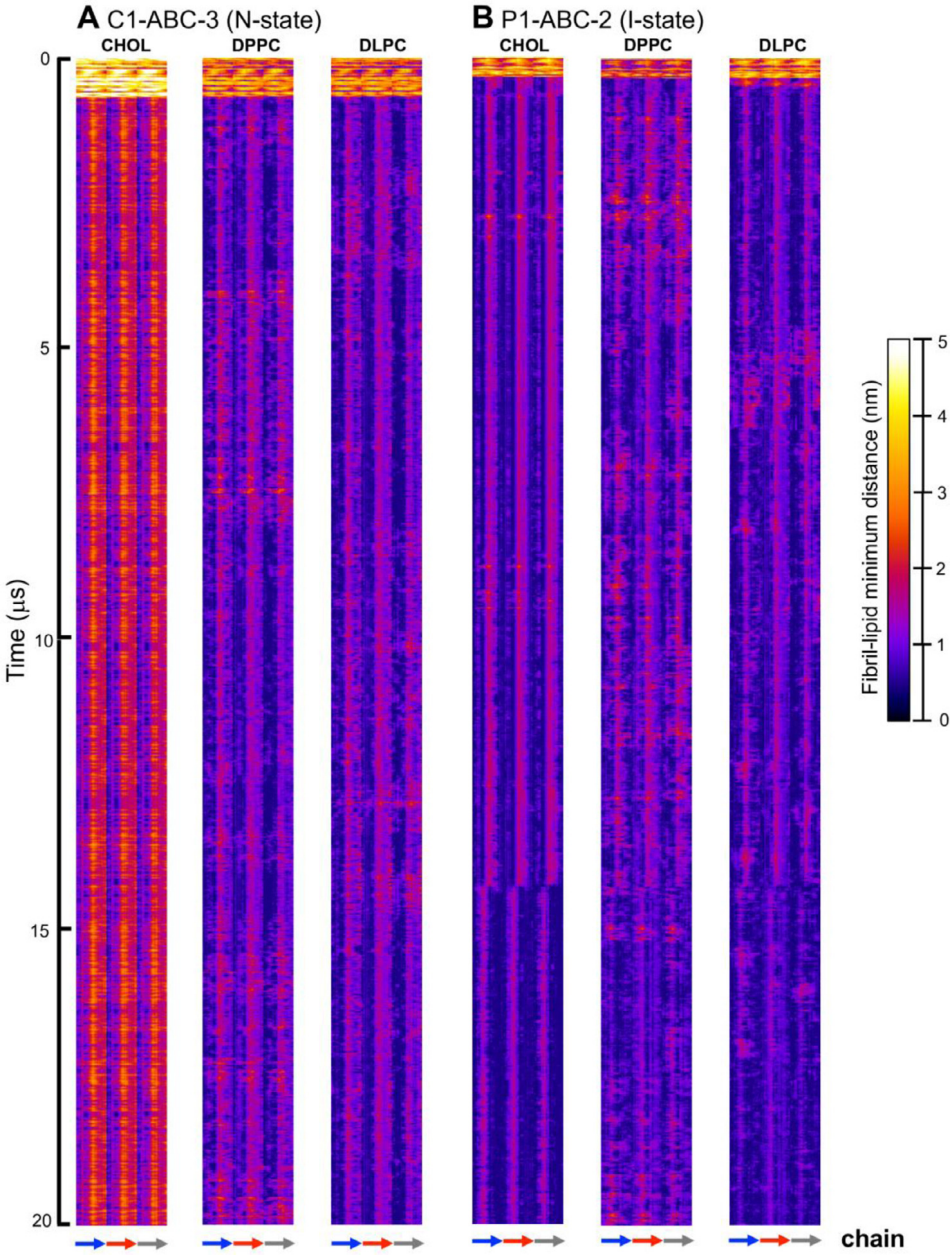
Fibril residue-lipid contact maps for C1-ABC-3 in N-state (A) and P1-ABC-2 in I-state (B) for CHOL, DPPC and DLPC. The chain residue (*x*-axis) and time (*y*-axis) are given. The color bar (0 to 5 nm) is also shown. The chain residues from N- to C-terminus of chains A (blue), B (red) and C (gray) are given in color arrows.

**Fig. 25:**
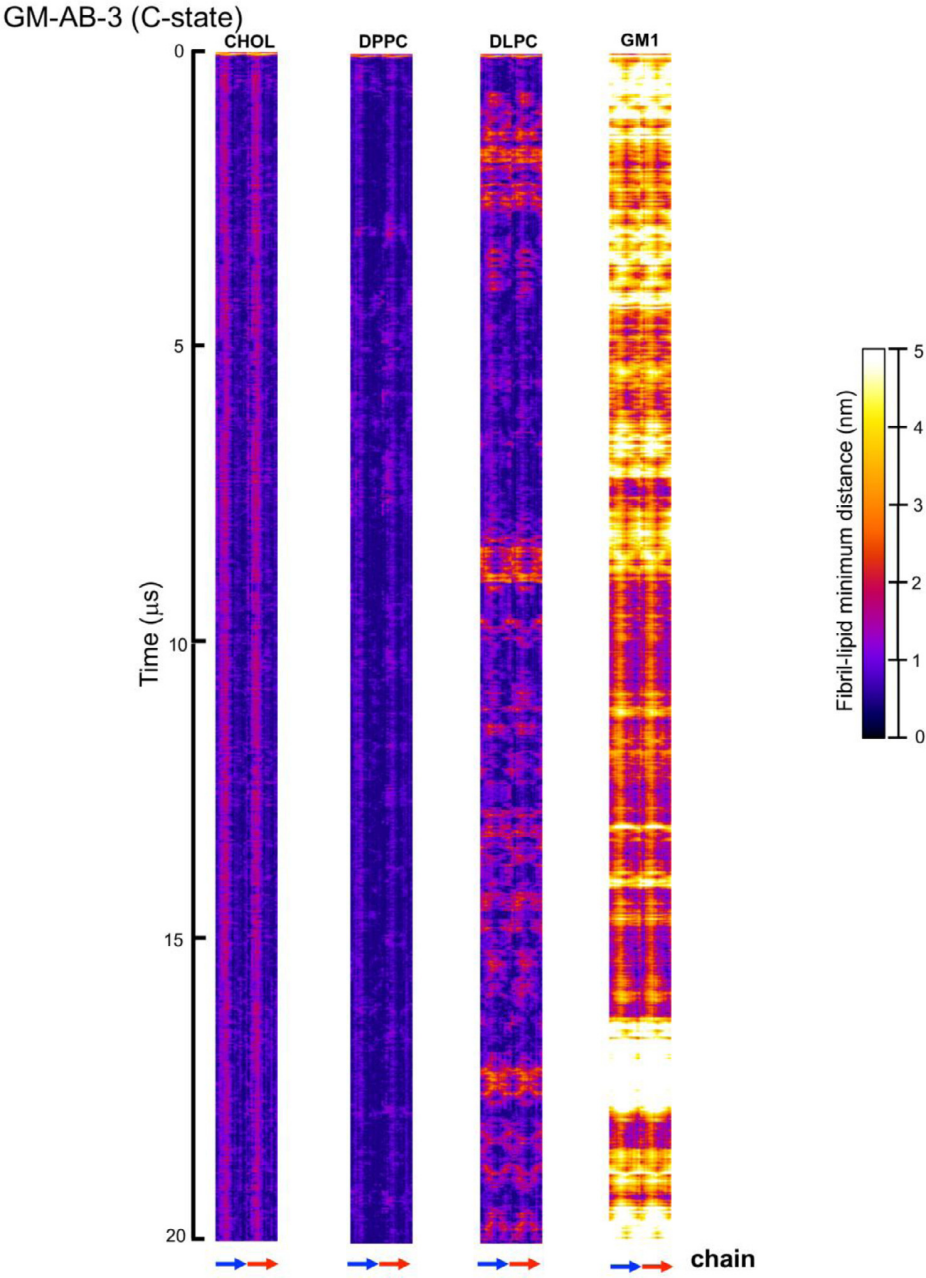
Fibril residue-lipid contact maps for GM-AB-3 in C-state for CHOL, DPPC, DLPC and GM1. The chain residue (*x*-axis) and time (*y*-axis) are given. The color bar (0–5 nm) is also shown. The chain residues from N- to C-terminus of chains A (blue) and B (red) are given in color arrows.

**Fig. 26:**
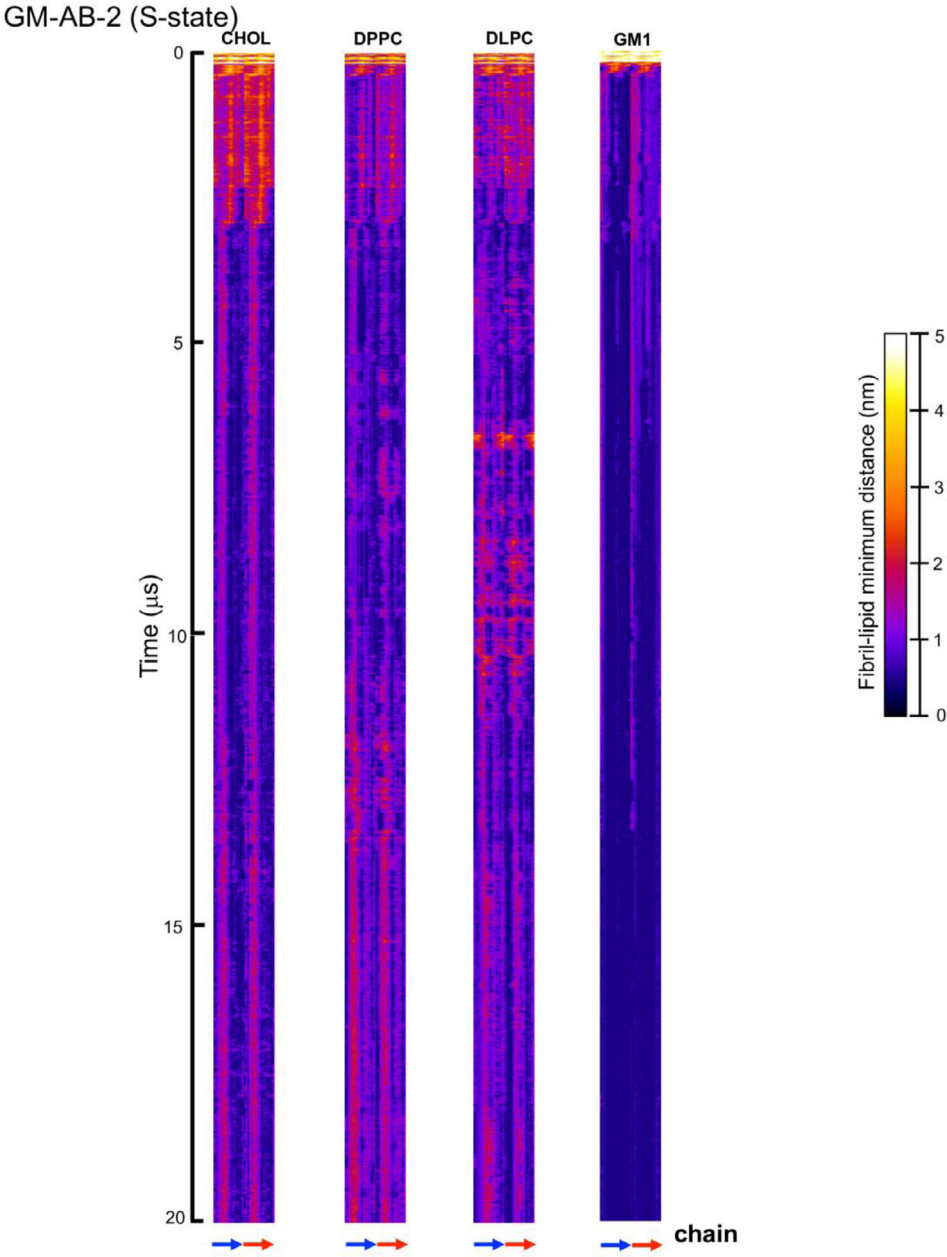
Fibril residue-lipid contact maps for GM-AB-2 in S-state for CHOL, DPPC, DLPC and GM1. The chain residue (*x*-axis) and time (*y*-axis) are given. The color bar (0–5 nm) is also shown. The chain residues from N- to C-terminus of chains A (blue) and B (red) are given in color arrows.

**Fig. 27:**
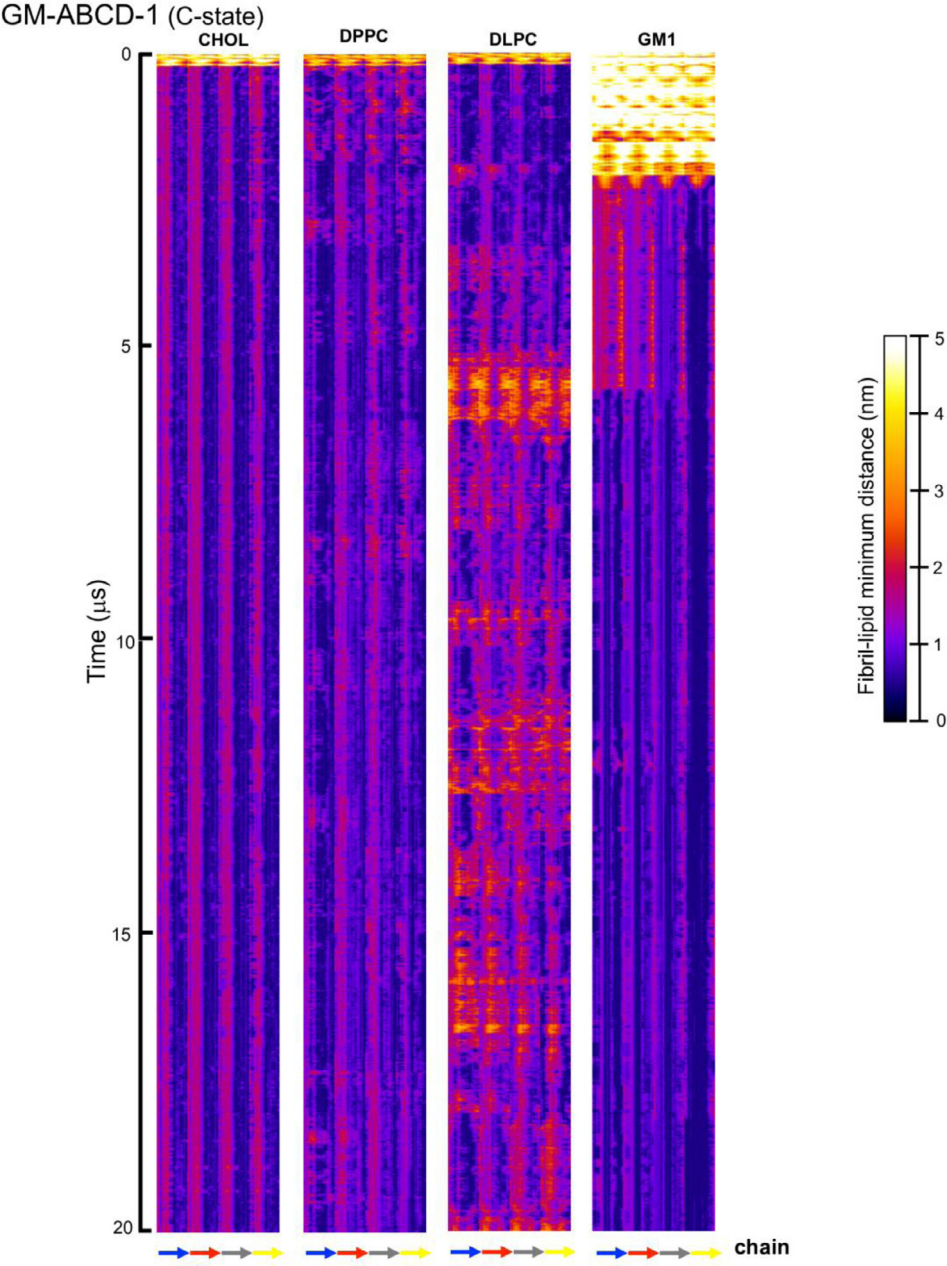
Fibril residue-lipid contact maps for GM-ABCD-1 in C-state for CHOL, DPPC, DLPC and GM1. The chain residue (*x*-axis) and time (*y*-axis) are given. The color bar (0 to 5 nm) is also shown. The chain residues from N- to C-terminus of chains A (blue), B (red), C (gray) and D (yellow) are given in color arrows.

**Fig. 28:**
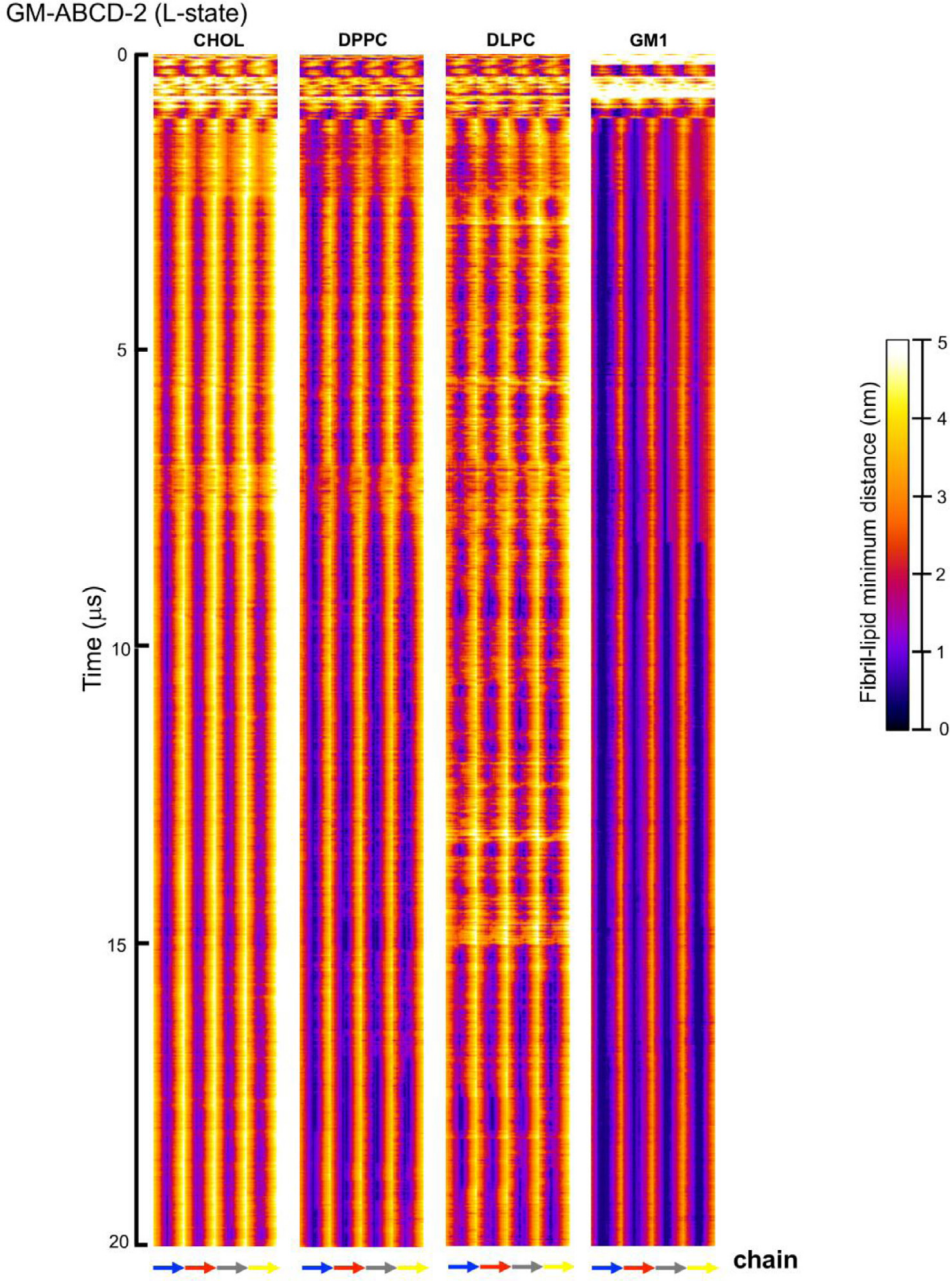
Fibril residue-lipid contact maps for GM-ABCD-2 in L-state for CHOL, DPPC, DLPC and GM1. The chain residue (*x*-axis) and time (*y*-axis) are given. The color bar (0–5 nm) is also shown. The chain residues from N- to C-terminus of chains A (blue), B (red), C (gray) and D (yellow) are given in color arrows.

Fig. 23, Fig. 24, Fig. 25, Fig. 26, Fig. 27, Fig. 28 show the fibril-lipid minimum distance (0–5 nm with color bar) vs. fibril residue number (horizontal or *x*-axis and in horizonal color arrows highlighting the fibril chains from N-terminus to C-terminus) and time (vertical or *y*-axis from 0 to 20 µs) for simulation replicates, CO-ABCD-2 in C-state, CO-ABCD-3 in T-state, C1-ABC-3 in N-state, P1-ABC-2 in I-state, GM-AB-3 in C-state, GM-AB-2 in S-state, GM-ABCD-1 in C-state and GM-ABCD-2 in L-state. All color maps (in PNG) are given in Supplementary Data (S23-28).

### 1.6 Fibril-lipid binding energy analysis

**Fig. 29:**
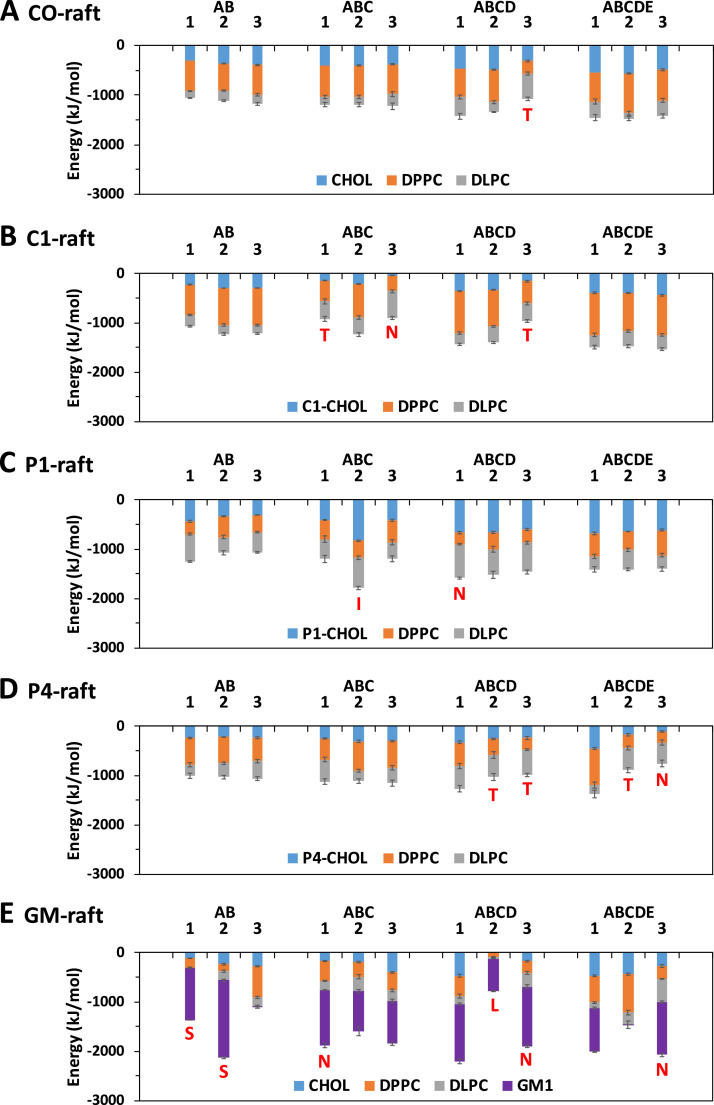
Time-averaged interaction energy between fibril and lipid (DPPC, DLPC, cholesterol and modified cholesterol and GM1) over the last 5 µs of the 0–20 µs simulation for fibrils of different sizes and replicates in fibril/raft complexes, CO-raft (A), C1-raft (B), P1-raft (C), P4-raft (D), and GM-raft (E). The uncertainty, SE of mean, is given. The membrane-bound states, other than the C-state, are labeled to facilitate the comparison.

Fig. 29 shows the time-averaged interaction energy between fibril and lipid over the last 5 µs of the simulations for all simulation replicates.

**Fig. 30:**
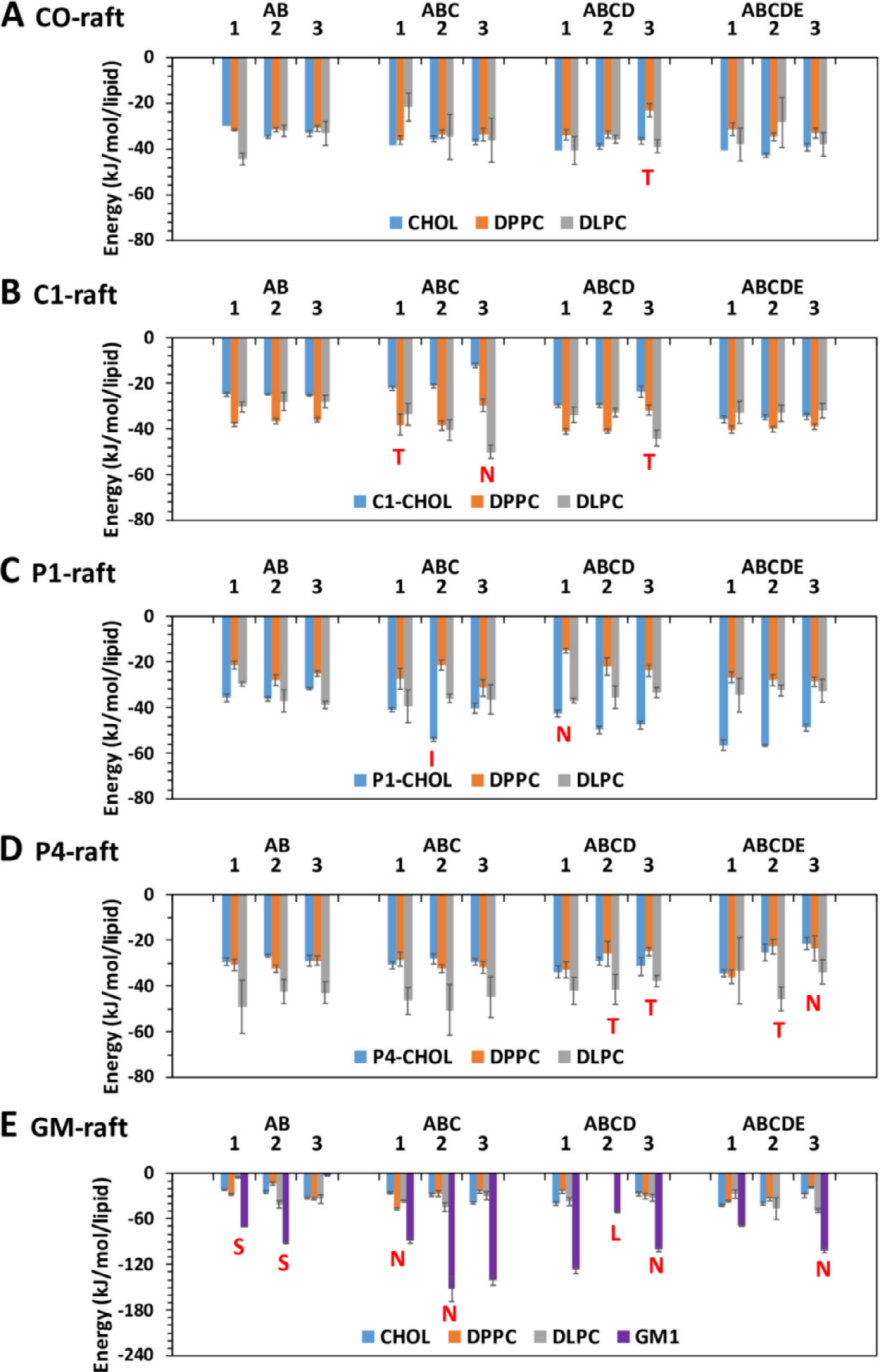
Time-averaged Interaction energy per lipid between fibril and lipid (DPPC, DLPC, cholesterol or modified cholesterol and GM1) over the last 5 µs of the 0–20 µs simulation for fibrils CO-raft (A), C1-raft (B), P1-raft (C), P4-raft (D), and GM-raft (E). Only the lipids within 0–10 Å are included in the energy calculations. See Fig. 29 legend for details.

Fig. 30 shows the time-averaged interaction energy/lipid between fibril and lipid over the last 5 µs of the simulations for all simulation replicates

**Fig. 31:**
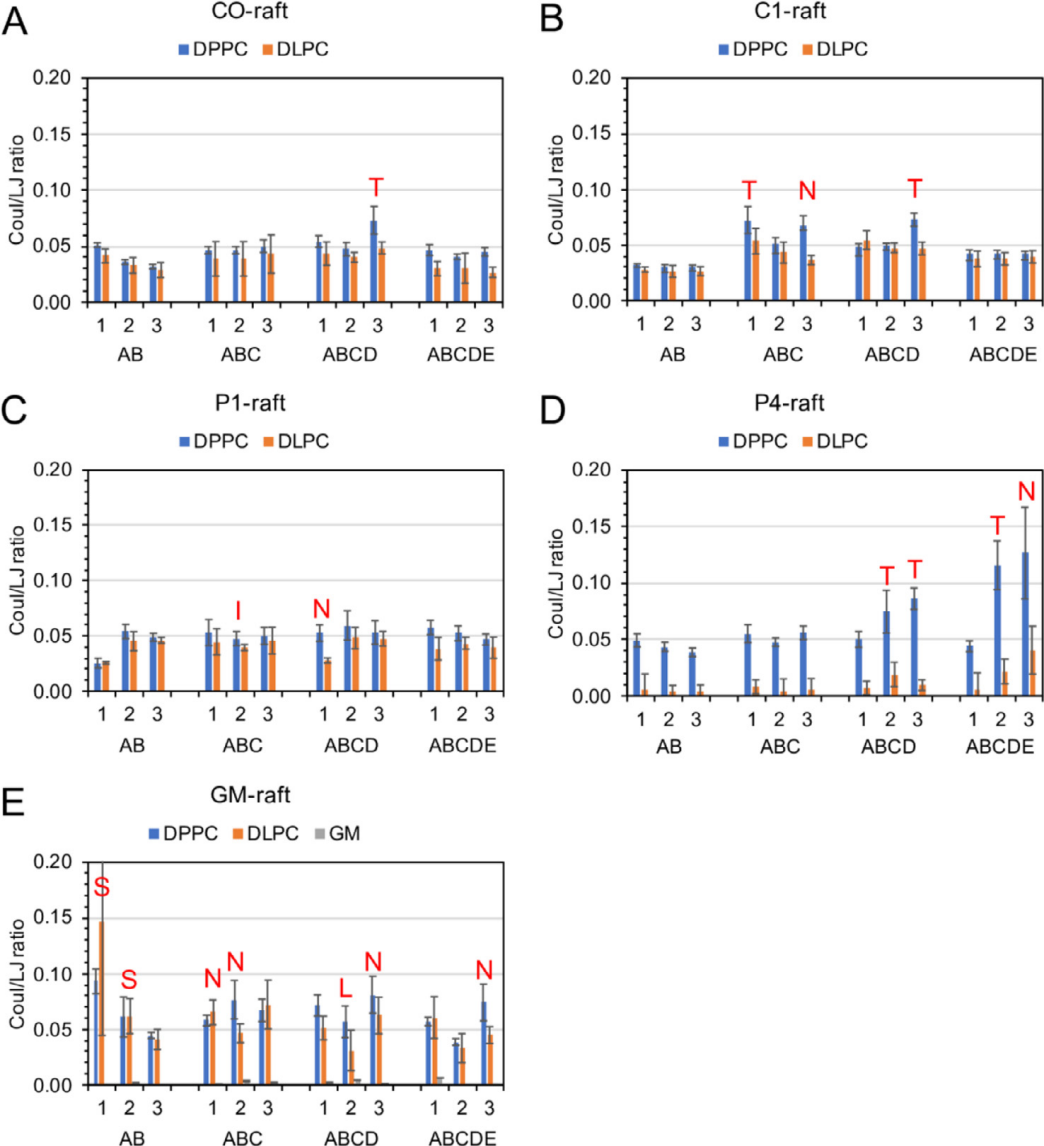
Time-averaged Coulomb and Lennard–Jones interaction energy (Coul/LJ) ratio between fibril and lipid (DPPC, DLPC, cholesterol and modified cholesterol, and GM1) over the last 5 µs of the 0–20 µs simulation for fibrils of different sizes and replicates in fibril/raft complexes, CO-raft (A), C1-raft (B), P1-raft (C), P4-raft (D), and GM-raft (E). The uncertainty, SE of mean, is given. The membrane-bound states, other than the C-state, are labeled to facilitate the comparison.

Fig. 31 shows the time-averaged Coulomb and Lennard-Jones interaction energy ratio between fibril and lipid over the last 5 µs of the simulations for all simulation replicates.

Data of the above binding energy calculations (in EXCEL) are given in Supplementary Data (S29–31).

### 1.7 Characterizations of annular lipid shell in lipid rafts

**Fig. 32:**
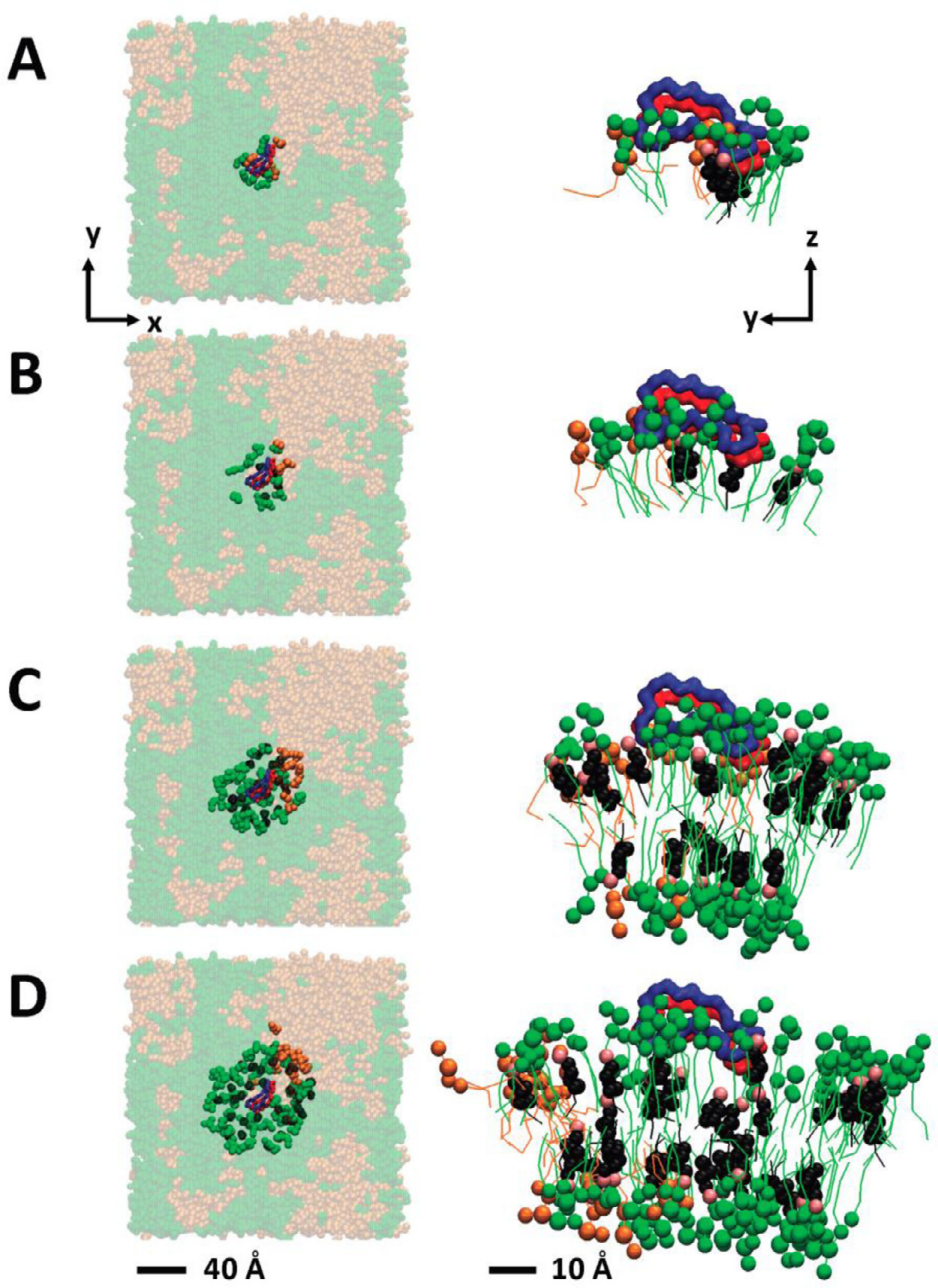
The lateral (*x*–*y*) and transverse (*y*–*z*) views of the four annular lipid shells of CO-raft based on the distances between the atoms of the membrane-bound fibril and lipids that satisfy the following criteria, less than 5 Å (A), 5 to 10 Å (B), 10 to 20 Å (C) and 20 to 30 Å (D), are shown. DPPC in green, DLPC in orange, CHO in black and dimer in blue and red ribbons are demonstrated. The scale bars for the lateral and transverse views are shown.

Fig. 32 shows the lateral and transverse views of four lipid shells of the simulation replicate CO-AB-2. The structural file of the replicate (in PDB) is given in Supplementary Data (S31).

**Fig. 33:**
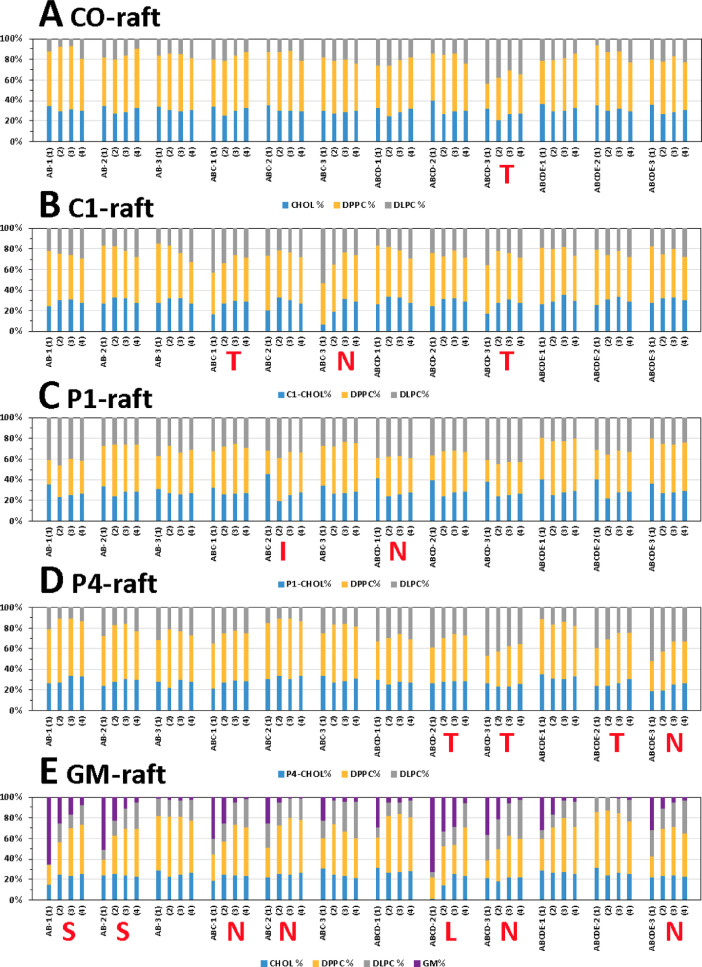
Time-averaged lipid composition of CHOL or modified CHOL (blue), DPPC (orange), DLPC (gray), and GM1 (purple) over the last 5 µs of the 0 to 20 µs simulation in four different annular lipid shells 1 to 4, according to their proximity from the protein, i.e., 5 Å (1), 5–10 Å (2), 10–20 Å (3), and 20–30 Å (4), in CO-raft (A), C1-raft (B), P1-raft (C), P4-raft (D) and GM-raft (E). The membrane-bound state (T, N, I, S or L) of each system are labeled by the corresponding red letter. The membrane-bound C-state is not labeled for clarity.

Fig. 33 shows the time-averaged lipid compositions of four annular lipid shells, based on the lipid proximity from the protein, i.e., 5 Å (shell 1), 5–10 Å (shell 2), 10–20 Å (shell 3) and 20–30 Å (shell 4), in all simulation replicates. The data (in EXCEL) are given in Supplementary Data (S33).

**Fig. 34:**
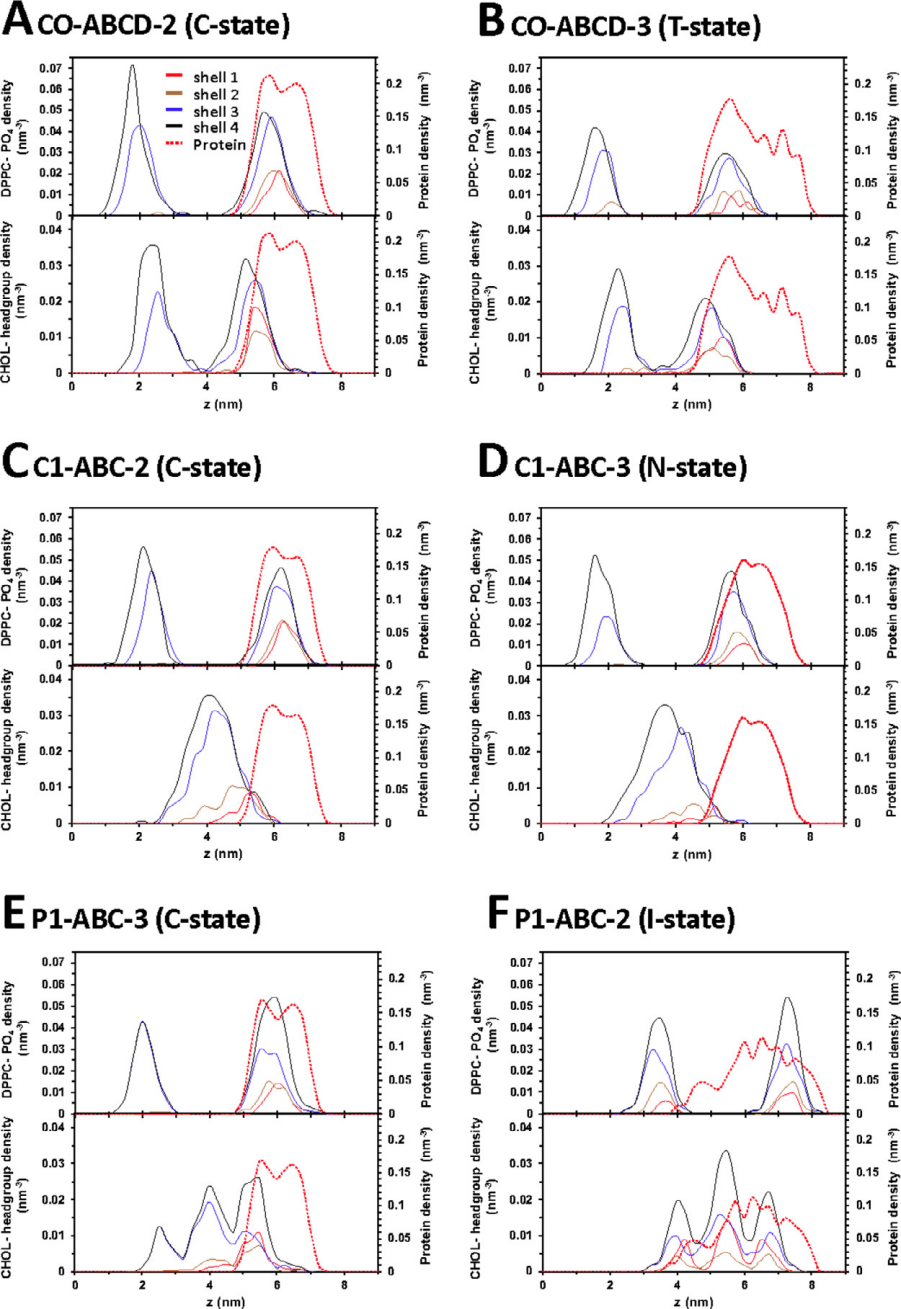
Representative time-averaged transverse number density of lipid atoms, DPPC-PO4 (upper panel) and CHOL-headgroup (lower panel), and protein (dashed red line in both panels) over the last 5 µs of the 0 to 20 µs simulation, in CO-ABCD-2 in C-state (A), CO-ABCD-3 in T-state (B), C1-ABC-2 in C-state (C), C1-ABC-3 in N-state (D), P1-ABC-3 in C-state (E) and P1-ABC-2 in I-state (F). The density distributions of lipids in different annular lipid shells 1 to 4, according to their proximity from the protein, i.e., 5 (red), 5–10 (brown), 10–20 (blue), and 20–30 Å (black), are shown. See Fig. 4 legend for details.

**Fig. 35:**
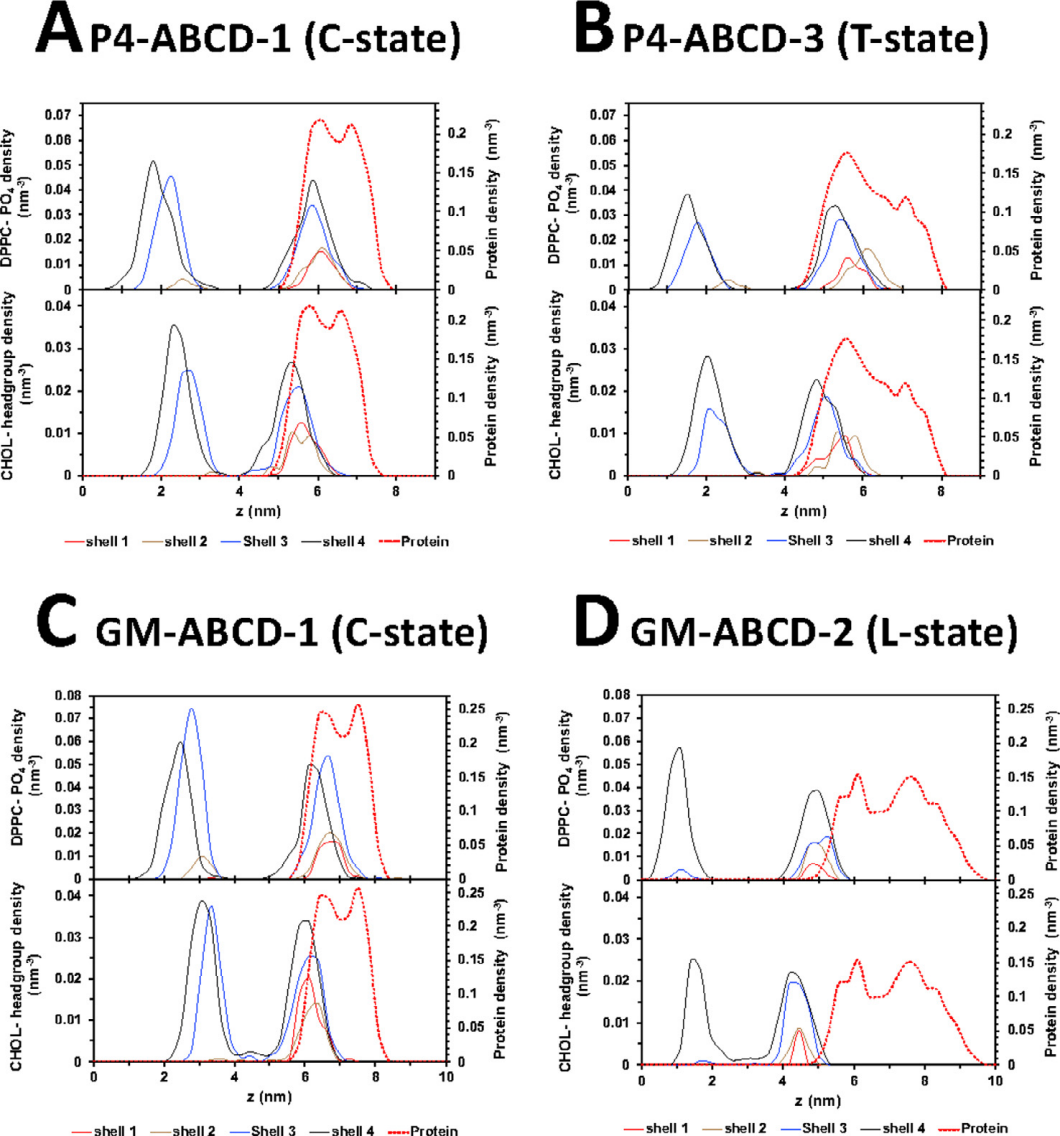
Representative time-averaged transverse number density of lipid atoms, DPPC-PO4 (upper panel) and CHOL-headgroup (lower panel), and protein (dashed red line in both panels) over the last 5 µs of the 0 to 20 µs simulation, in P4-ABCD-1 in the C-state (A), P4-ABCD-3 in the T-state (B), GM-ABCD-1 in the C-state (C), GM-ABCD-2 in the L-state (D) are shown. See the legend of Fig. 20 for details of lipid shell identification.

Figs. 34 and 35 show the representative time-averaged transverse number density of lipid atoms, DPPC phosphate and CHOL headgroup, and protein vs. distance along the z-axis of lipids in four annular lipid shells. All data (in EXCEL) are given in Supplementary Data (S34-35)

**Fig. 36:**
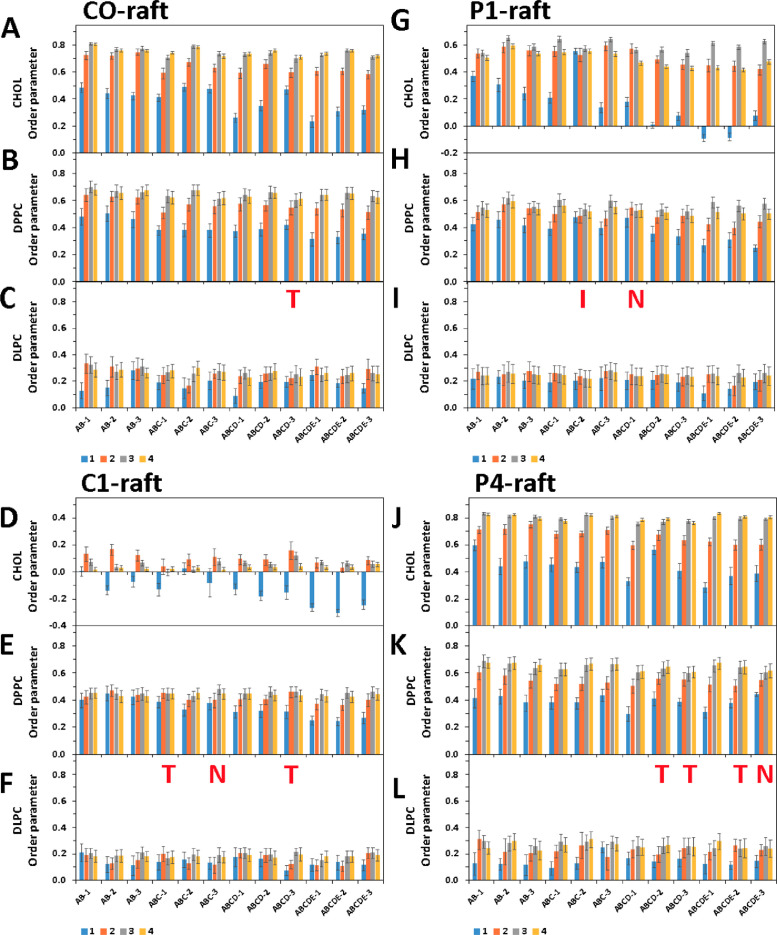
Time-averaged order parameter of lipids (cholesterol, DPPC and DLPC) over the last 5 µs of the 0 to 20 µs simulation in all four annular lipid shell of fibril/raft complexes, CO-raft (A)–(C), C1-raft (G)–(I), P1-raft (D)–(F), and P4-raft (J)–(L) of different sizes and replicates are shown. The uncertainty, SE of mean, is given. The membrane-bound state (T, N, I, S or L) of each system are labeled by the corresponding red letter. The membrane-bound C-state is not labeled for clarity.

Fig. 36 shows the time-averaged lipid order parameters in four annular lipid shells over the last 5 µs of simulations in CO-raft, C1-raft, P1-raft and GM-raft. All data (in EXCEL) are given in Supplementary Data (S36)

**Fig. 37:**
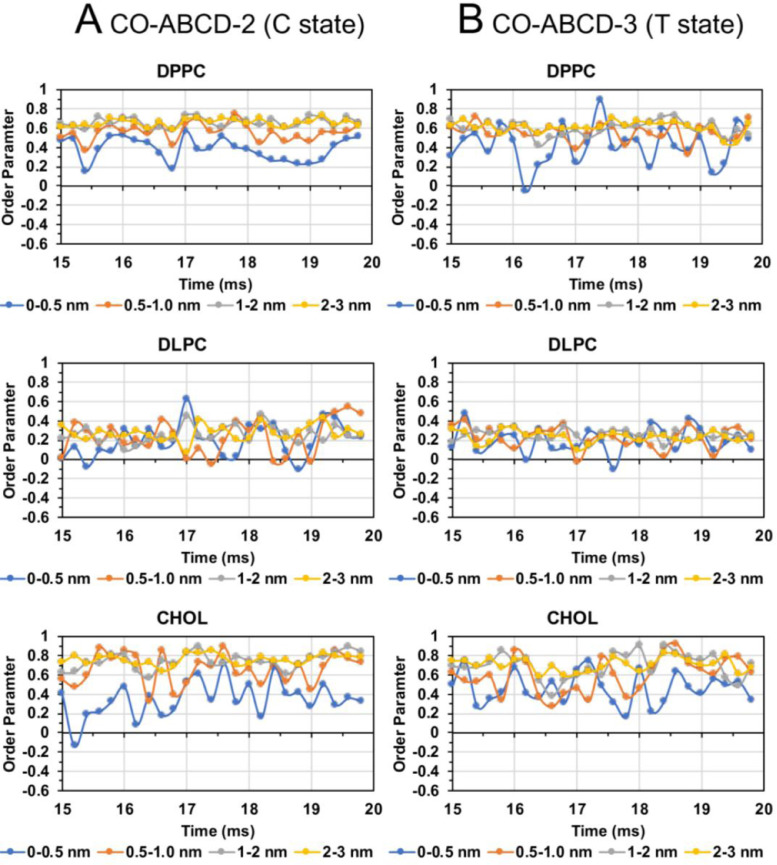
Lipid order parameter of DPPC (upper panel), DLPC (mid panel) and CHOL (lower panel) vs. time in four annular lipid shells, 0–0.5 (blue), 0.5–1.0 (orange), 1–2 (gray) and 2–3 nm (yellow), for CO-ABCD-2 in C-state (A) and CO-ABCD-3 in T-state (B).

**Fig. 38:**
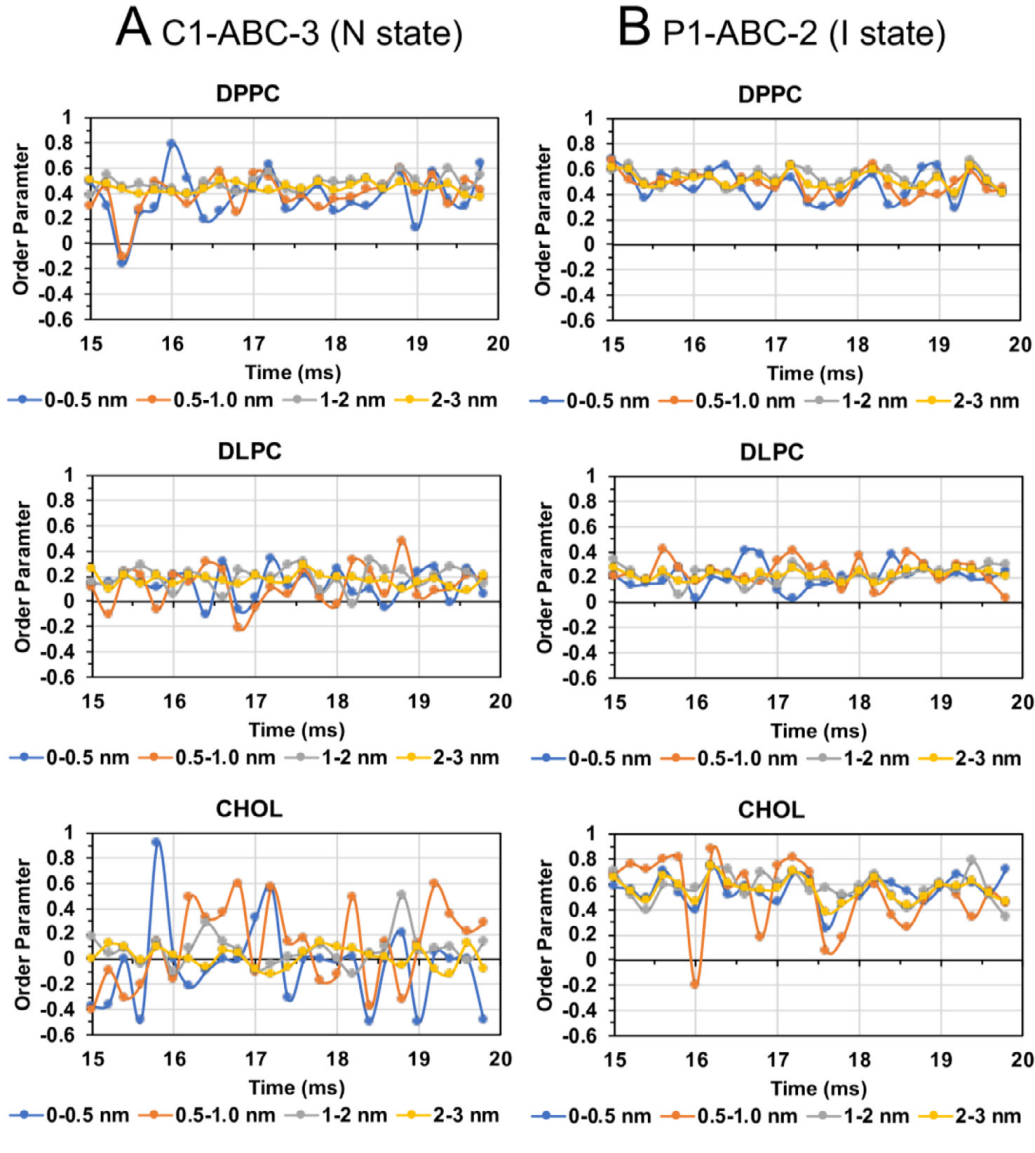
Lipid order parameter of DPPC (upper panel), DLPC (mid panel) and CHOL (lower panel) vs. time in four annular lipid shells, 0–0.5 (blue), 0.5–1.0 (orange), 1–2 (gray) and 2–3 nm (yellow), for C1-ABC-3 in N-state (A) and P1-ABC-2 in I-state (B).

**Fig. 39:**
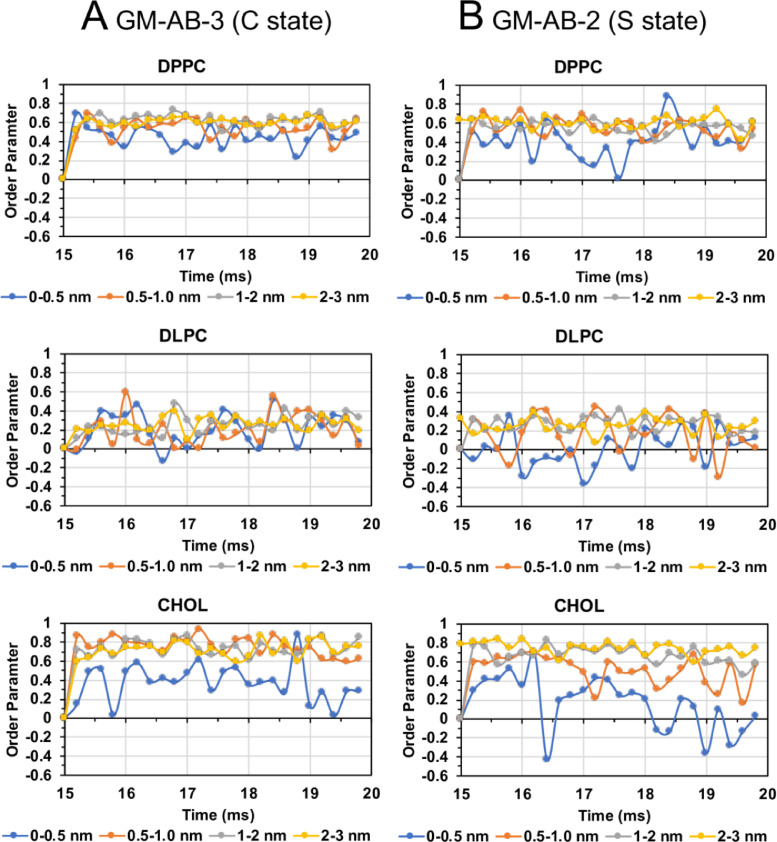
Lipid order parameter of DPPC (upper panel), DLPC (mid panel) and CHOL (lower panel) vs. time in four annular lipid shells, 0–0.5 (blue), 0.5–1.0 (orange), 1–2 (gray) and 2–3 nm (yellow), for GM-AB-3 in C-state (A) and GM-AB-2 in S-state (B).

**Fig. 40:**
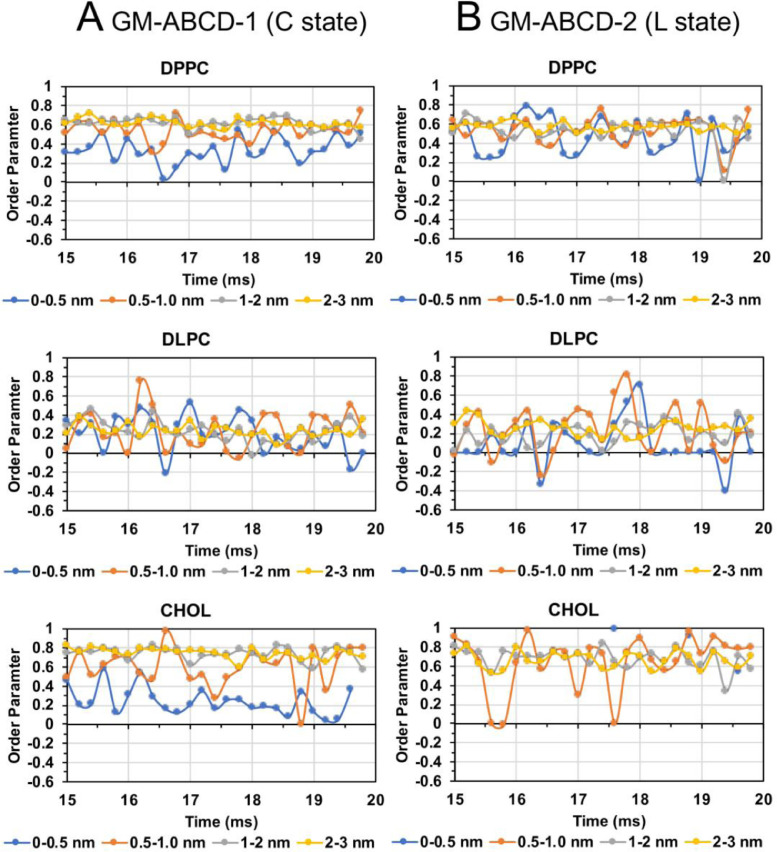
Lipid order parameter of DPPC (upper panel), DLPC (mid panel) and CHOL (lower panel) vs. time in four annular lipid shells, 0–0.5 (blue), 0.5–1.0 (orange), 1–2 (gray) and 2–3 nm (yellow), for GM-ABCD-1 in C-state (A) and GM-ABCD-2 in l-state (B).

Fig. 37, Fig. 38, Fig. 39, Fig. 40 shows the time evolutions of lipid order parameters in four annular lipid shells of CO-ABCD-2 in C-state, CO-ABCD-3 in T-state, C1-ABC-3 in N-state, P1-ABC-2 in I-state, GM-AB-3 in C-state, GM-AB-2 in S-state, GM-ABCD-1 in C-state and GM-ABCD-2 in L-state. All data (in EXCEL) are given in Supplementary Data (S37-40).

## 2 Experimental design, materials, and methods

The four ternary lipid rafts (CO-raft, C1-raft, P1-raft and P4-raft) were designed and constructed based on the pre-equilibrated ternary lipid raft reported by Risselada and Marrink [9]. It also represents the control lipid raft (CO-raft). The C1-raft (DPPC/DLPC/C1-CHOL) was constructed by modifying the polarity of the CHOL headgroup from a polar type (SP1) to a non-polar type (C1), i.e., CHOL modified to C1-CHOL. The P1-raft (DPPC/DLPC/P1-CHOL) was constructed by modifying the polarity of the CHOL tail-group from a non-polar type (C1) to a polar type (P1), i.e., CHOL modified to P1-CHOL. The P4-raft (DPPC/DLPC/P4-CHOL) was constructed by modifying the polarity of the CHOL headgroup from a polar type (SP1) to a more polar type (P4), i.e., CHOL modified to P4-CHOL. The polarity of the coarse-grained atom, i.e., SP1, C1, P1 or P4, is based on the published Martini Force Fields of lipids [2]. The lipid molar ratio of DPPC: DLPC:CHOL, DPPC:DLPC:C1-CHOL, DPPC:DLPC:P1-CHOL or DPPC:DLPC:P4-CHOL is 0.42:0.28:0.30 in CO-raft, C1-raft, P1-raft or P4-raft, respectively. Each raft has ∼ 36,000 water, 828 DPPC, 540 DLPC and 576 CHOL, C1-CHOL, P1-CHOL or P4-CHOL, and in 0.1 M of NaCl.

The asymmetric and quaternary GM-raft (DPPC/DLPC/CHOL/GM1) was constructed by replacing some DPPC, DLPC and CHOL on one lipid monolayer with GM1-lipid based on the CO-raft. The lipid molar ratio of GM-raft is 0.02:0.43:0.30:0.25. This raft has ∼ 36,000 waters, 36 GM1, 709 DPPC, 487 DLPC and 410 CHOL, and in 0.1 M of NaCl.

Each of the five lipid rafts underwent energy minimization and 2 ns pre-equilibration under position-restraint on all lipid and protein atoms to allow proper hydration before the production, removal of position restraints, molecular dynamics simulations for 20 microseconds. The detailed design, construction and simulation procedures based on Martini Coarse-grained force fields [2] and GROMACS [3] are given in the research article [1].

Data analysis involves lipid-selection, orientational parameter of lipid and protein, minimum-distance analysis and molecular visualization. The use of data-filtering tool, *g_select*, from GROMACS [3], to select DPPC-rich Lo-domain, DLPC-rich Ld-domain and mixed Lo/Ld or Lod domain, as well as annular lipid shells, based on the proximity of the lipid and protein atoms upon fibril binding to the lipid membranes. The *g_order* tool from GROMACS [3] was used to calculate the segmental orientation order of lipid acyl chains and protein fibril chains in each lipid domain or annular lipid shell. The *g_mind* tool from GROMACS [3] was used to determine the minimum-distance between the atoms of lipid and fibril vs. time, number of atom contacts between lipid and fibril vs. time and the time-averaged minimum-distance between lipid and fibril vs. fibril residue number. The membrane binding time, i.e., the time that the fibril first establishes close contacts to the membrane surface and stays on membrane surface, was determined by the 2D fibril-lipid minimum distance vs. time plots (Figs. 17–21), and by the 3D minimum-distance vs. fibril residue vs. time plots (Figs. 24–28). Finally, both the membrane binding time and the stability of the membrane-bound state was determined by the 3D protein orientation vs. residue vs. time plots (Figs. 13 and 14). The molecular visualization of lipid domains and annular lipid shells were also performed using the *representation selection tool* in Visual Molecular Dynamics program (VMD) [10].

## Appendix Supplementary materials

Image, application 1
