# Supplementary material for: Data showing the lipid conformations and membrane binding behaviors of beta-amyloid fibrils in phase-separated cholesterol-enriched lipid domains with and without glycolipid and oxidized cholesterol from coarse-grained molecular dynamics simulations
Source: Data Brief. 2020 Apr 19;30:105496. doi: 10.1016/j.dib.2020.105496 (PMC7186522; doi:10.1016/j.dib.2020.105496)
Supplement: Supplementary file 1 [file mmc1.zip › Supplementary-Data/TableS1-A-C.docx]

**Table S1a.** Membrane binding times and membrane bound-orientations of fibrils in CO-raft and C1-raft.

| Simulation replicate | Binding time (μs) | | | Binding conformation | |
| --- | --- | --- | --- | --- | --- |
| (0-20μs) | DPPC | CHOL | DLPC | Lipid layer | State |
|  |  |  |  |  |  |
| CO-AB-1 | 0.365 | 0.365 | 0.348 | Lower | C |
| CO-AB-2 | 0.005 | 0.005 | 0.005 |  | C |
| CO-AB-3 | 0.280 | 0.280 | 0.277 |  | C |
| CO-ABC-1 | 0.265 | 0.265 | 0.253 | Lower | C |
| CO-ABC-2 | 0.221 | 0.221 | 0.221 | Lower | C |
| CO-ABC-3 | 1.330 | 1.330 | 1.325 |  | C |
| CO-ABCD-1 | 0.889 | 0.889 | 1.088 |  | C |
| CO-ABCD-2 | 4.210 | 4.270 | 4.210 | Lower | C |
| CO-ABCD-3 | 0.439 | 0.451 | 0.430 |  | **T** |
| CO-ABCDE-1 | 0.451 | 0.451 | 0.442 |  | C |
| CO-ABCDE-2 | 0.348 | 0.348 | 0.348 | Lower | C |
| CO-ABCDE-3 | 0.196 | 0.275 | 0.277 | Lower | C |
|  |  |  |  |  |  |
| C1-AB-1 | 0.600 | 0.607 | 0.600 |  | C |
| C1-AB-2 | 0.346 | 0.377 | 0.346 |  | C |
| C1-AB-3 | 0.139 | 0.139 | 0.139 |  | C |
| C1-ABC-1 | 0.140 | 0.160 | 0.140 | Lower | **T** |
| C1-ABC-2 | 0.306 | 0.313 | 0.302 |  | C |
| C1-ABC-3 | 0.690 | 0.700 | 0.660 | Lower | **N** |
| C1-ABCD-1 | 0.331 | 0.355 | 0.321 |  | C |
| C1-ABCD-2 | 0.856 | 0.856 | 0.842 | Lower | C |
| C1-ABCD-3 | 0.168 | 0.196 | 0.168 | Lower | **T** |
| C1-ABCDE-1 | 2.460 | 2.470 | 2.460 | Lower | C |
| C1-ABCDE-2 | 0.340 | 0.342 | 0.340 | Lower | C |
| C1-ABCDE-3 | 0.280 | 0.292 | 0.280 | Lower | C |

Fibril membrane binding times from fibril-lipid minimum distance kinetics of each simulation replicate is shown. Information about the fibril binding conformation, in terms of the location of fibril on lipid bilayer (upper or lower leaflet) and the membrane-bound state, for each simulation replicate is also presented. Starting from the solution phase, each fibril attached to either the upper or lower leaflet of the lipid bilayer after the 20 μs simulation, of each fibril/raft complex. For clarity, only the lower lipid layer binding events are identified below, if not indicated the fibril bound to the upper lipid layer. Membrane bound states other than C-states are indicated in bold.

**Table S1b.** Membrane binding times and membrane bound-orientations of fibrils in P1-raft and P4-raft.

| Simulation replicate | Binding Time (μs) | | | Binding conformation | |
| --- | --- | --- | --- | --- | --- |
| (0-20μs) | DPPC | CHOL | DUPC | Lipid layer | State |
|  |  |  |  |  |  |
| P1-AB-1 | 0.237 | 0.237 | 0.237 | Lower | C |
| P1-AB-2 | 0.422 | 0.422 | 0.418 | Lower | C |
| P1-AB-3 | 0.317 | 0.317 | 0.317 | Lower | C |
| P1-ABC-1 | 0.377 | 0.377 | 0.377 | Lower | C |
| P1-ABC-2 | 0.331 | 0.334 | 0.333 | Lower | **I** |
| P1-ABC-3 | 0.060 | 0.060 | 0.070 | Lower | C |
| P1-ABCD-1 | 2.760 | 2.777 | 2.760 |  | **N** |
| P1-ABCD-2 | 0.123 | 0.125 | 0.123 | Lower | C |
| P1-ABCD-3 | 1.310 | 1.310 | 1.310 | Lower | C |
| P1-ABCDE-1 | 0.629 | 0.629 | 0.629 |  | C |
| P1-ABCDE-2 | 1.416 | 1.422 | 1.416 | Lower | C |
| P1-ABCDE-3 | 0.640 | 0.640 | 0.640 |  | C |
|  |  |  |  |  |  |
|  |  |  |  |  |  |
| P4-AB-1 | 2.020 | 2.000 | 1.900 | Lower | C |
| P4-AB-2 | 0.145 | 0.145 | 0.192 |  | C |
| P4-AB-3 | 0.421 | 0.421 | 0.416 | Lower | C |
| P4-ABC-1 | 1.098 | 1.089 | 1.089 | Lower | C |
| P4-ABC-2 | 1.062 | 1.065 | 1.052 |  | C |
| P4-ABC-3 | 0.690 | 0.693 | 0.684 |  | C |
| P4-ABCD-1 | 0.957 | 0.957 | 0.948 | Lower | C |
| P4-ABCD-2 | 0.482 | 0.500 | 0.441 |  | **T** |
| P4-ABCD-3 | 1.660 | 1.660 | 1.510 |  | **T** |
| P4-ABCDE-1 | 0.075 | 0.075 | 0.075 |  | C |
| P4-ABCDE-2 | 2.240 | 2.245 | 2.240 | Lower | **T** |
| P4-ABCDE-3 | 3.270 | 3.270 | 3.270 | Lower | **N** |
|  |  |  |  |  |  |

Fibril membrane binding time from fibril-lipid minimum distance kinetics of each simulation replicate is shown. Also, information of the fibril binding conformation, in terms of the location of fibril on lipid bilayer (upper or lower leaflet) and the membrane-bound state, for each simulation replicate is presented. See figure caption of Table S1a for description of table formatting.

**Table S1c.** Membrane binding times and bound-orientations of fibrils in fibril/GM-raft complexes.

| Simulation replicate | Binding Time (μs) | | | | Binding conformation | |
| --- | --- | --- | --- | --- | --- | --- |
| (0-20μs) | DPPC | CHOL | DLPC | GM1 | Lipid layer | State |
|  |  |  |  |  |  |  |
| GM-AB-1 | 0.626 | 0.679 | 0.626 | 0.377 |  | **S** |
| GM-AB-2 | 2.296 | 2.296 | 2.296 | 0.244 |  | **S** |
| GM-AB-3 | 0.059 | 0.059 | 0.059 |  | Lower | C |
| GM-ABC-1 | 0.589 | 0.589 | 0.521 | 0.290 |  | **N** |
| GM-ABC-2 | 0.936 | 0.948 | 0.936 | 5.840 |  | **N** |
| GM-ABC-3 | 0.513 | 0.513 | 0.513 | 0.498 |  | C |
| GM-ABCD-1 | 0.189 | 0.193 | 0.189 | 2.080 |  | C |
| GM-ABCD-2 |  |  |  | 0.981 |  | **L** |
| GM-ABCD-3 | 1.080 | 1.080 | 1.080 | 0.424 |  | **N** |
| GM-ABCDE-1 | 0.805 | 0.812 | 0.805 | 0.612 |  | C |
| GM-ABCDE-2 | 0.122 | 0.127 | 0.122 |  | Lower | C |
| GM-ABCDE-3 | 9.120 | 9.140 | 9.120 | 0.388 |  | **N** |
|  |  |  |  |  |  |  |

Fibril membrane binding time from fibril-lipid minimum distance kinetics of each simulation replicate is shown. Also, information of the fibril binding conformation, in terms of the location of fibril on lipid bilayer (upper or lower leaflet) and the membrane-bound state, for each simulation replicate is presented. See figure caption of Table S1a for description of table formatting..
