# Supplementary figures and images for: Data showing the lipid conformations and membrane binding behaviors of beta-amyloid fibrils in phase-separated cholesterol-enriched lipid domains with and without glycolipid and oxidized cholesterol from coarse-grained molecular dynamics simulations
Source: Data Brief. 2020 Apr 19;30:105496. doi: 10.1016/j.dib.2020.105496 (PMC7186522; doi:10.1016/j.dib.2020.105496)

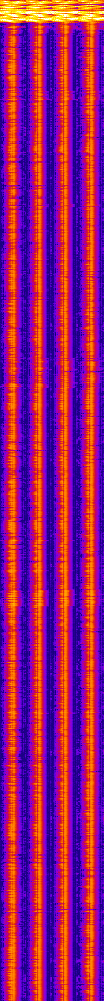

Supplement: Supplementary file 1 [file mmc1.zip › Supplementary-Data/S23-28/Fig.S23B-CO-ABCD-3-CHO-output_matrix.png]

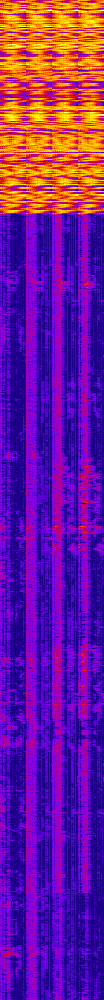

Supplement: Supplementary file 1 [file mmc1.zip › Supplementary-Data/S23-28/Fig.S23A-CO-ABCD-2-DPP-output_matrix.png]

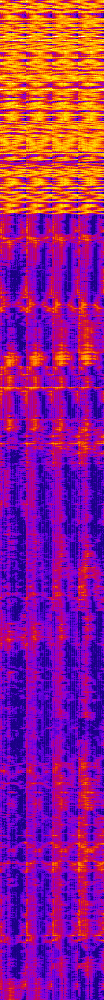

Supplement: Supplementary file 1 [file mmc1.zip › Supplementary-Data/S23-28/Fig.S23A-CO-ABCD-2-DLP-output_matrix.png]

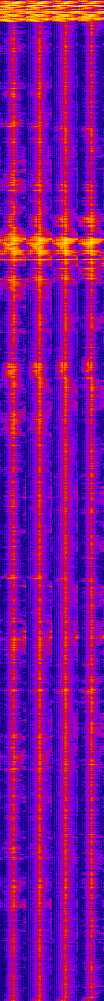

Supplement: Supplementary file 1 [file mmc1.zip › Supplementary-Data/S23-28/Fig.S23B-CO-ABCD-3-DLP-output_matrix.png]

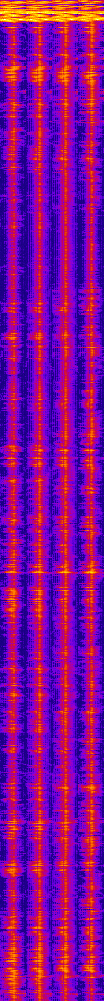

Supplement: Supplementary file 1 [file mmc1.zip › Supplementary-Data/S23-28/Fig.S23B-CO-ABCD-3-DPP-output_matrix.png]

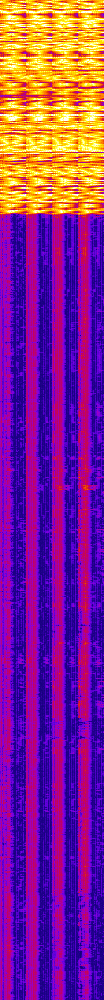

Supplement: Supplementary file 1 [file mmc1.zip › Supplementary-Data/S23-28/Fig.S23A-CO-ABCD-2-CHO-output_matrix.png]

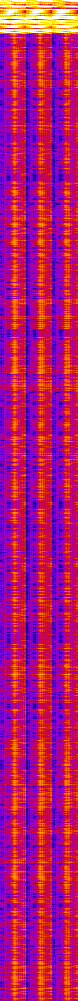

Supplement: Supplementary file 1 [file mmc1.zip › Supplementary-Data/S23-28/Fig.S24A-C1-ABC-3-CHO-output_matrix.png]

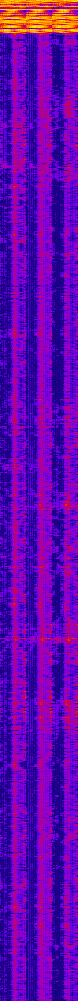

Supplement: Supplementary file 1 [file mmc1.zip › Supplementary-Data/S23-28/Fig.S24A-C1-ABC-3-DLP-output_matrix.png]

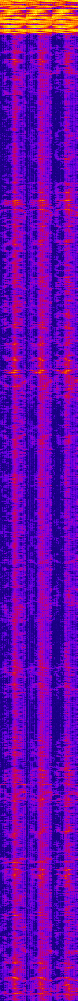

Supplement: Supplementary file 1 [file mmc1.zip › Supplementary-Data/S23-28/Fig.S24A-C1-ABC-3-DPP-output_matrix.png]

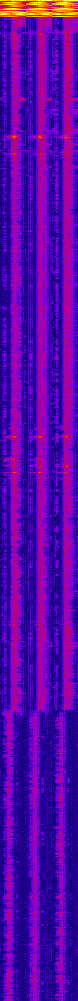

Supplement: Supplementary file 1 [file mmc1.zip › Supplementary-Data/S23-28/Fig.S24B-P1-ABC-2-CHO-output_matrix.png]

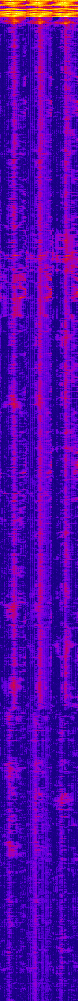

Supplement: Supplementary file 1 [file mmc1.zip › Supplementary-Data/S23-28/Fig.S24B-P1-ABC-2-DLP-output_matrix.png]

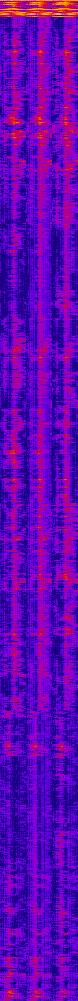

Supplement: Supplementary file 1 [file mmc1.zip › Supplementary-Data/S23-28/Fig.S24A-P1-ABC-2-DPP-output_matrix.png]

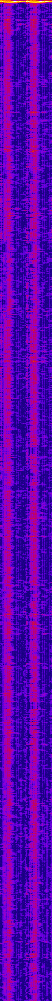

Supplement: Supplementary file 1 [file mmc1.zip › Supplementary-Data/S23-28/Fig.S25-GM-AB-3-CHO-output_matrix.png]

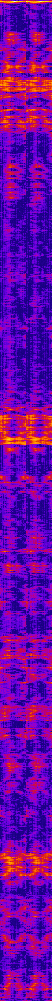

Supplement: Supplementary file 1 [file mmc1.zip › Supplementary-Data/S23-28/Fig.S25-GM-AB-3-DLP-output_matrix.png]

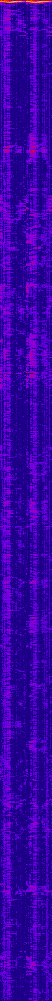

Supplement: Supplementary file 1 [file mmc1.zip › Supplementary-Data/S23-28/Fig.S25-GM-AB-3-DPP-output_matrix.png]

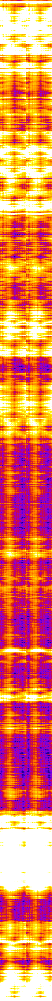

Supplement: Supplementary file 1 [file mmc1.zip › Supplementary-Data/S23-28/Fig.S25-GM-AB-3-GM-output_matrix.png]

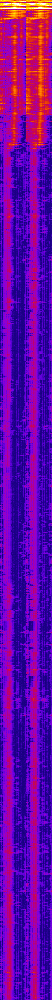

Supplement: Supplementary file 1 [file mmc1.zip › Supplementary-Data/S23-28/Fig.S26-GM-AB-CHO-2-output_matrix.png]

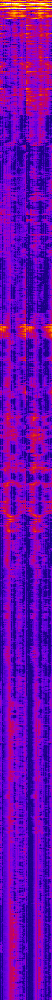

Supplement: Supplementary file 1 [file mmc1.zip › Supplementary-Data/S23-28/Fig.S26-GM-AB-DLP-2-output_matrix.png]

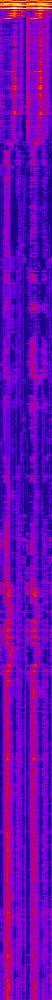

Supplement: Supplementary file 1 [file mmc1.zip › Supplementary-Data/S23-28/Fig.S26-GM-AB-DPP-2-output_matrix.png]

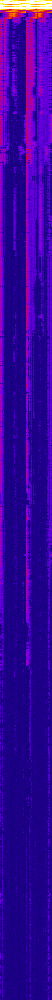

Supplement: Supplementary file 1 [file mmc1.zip › Supplementary-Data/S23-28/Fig.S26-GM-AB-GM-2-output_matrix.png]

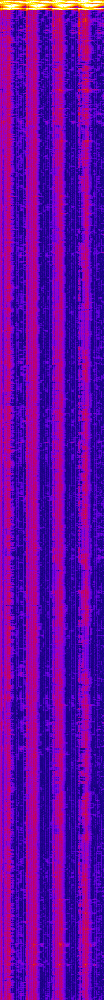

Supplement: Supplementary file 1 [file mmc1.zip › Supplementary-Data/S23-28/Fig.S27-GM-ABCD-1-CHO-output_matrix.png]

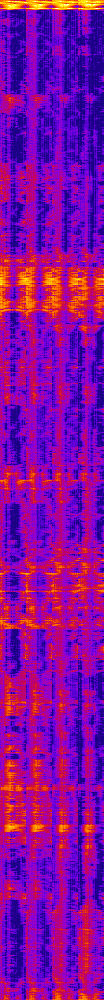

Supplement: Supplementary file 1 [file mmc1.zip › Supplementary-Data/S23-28/Fig.S27-GM-ABCD-1-DLP-output_matrix.png]

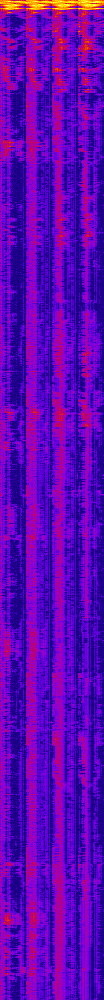

Supplement: Supplementary file 1 [file mmc1.zip › Supplementary-Data/S23-28/Fig.S27-GM-ABCD-1-DPP-output_matrix.png]

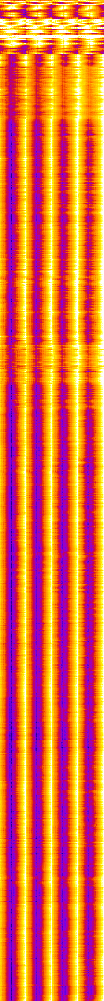

Supplement: Supplementary file 1 [file mmc1.zip › Supplementary-Data/S23-28/Fig.S28-GM-ABCD-2-CHO-output_matrix.png]

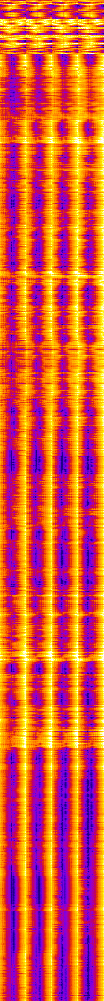

Supplement: Supplementary file 1 [file mmc1.zip › Supplementary-Data/S23-28/Fig.S28-GM-ABCD-2-DLP-output_matrix.png]

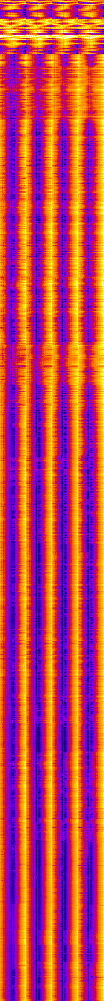

Supplement: Supplementary file 1 [file mmc1.zip › Supplementary-Data/S23-28/Fig.S28-GM-ABCD-2-DPP-output_matrix.png]

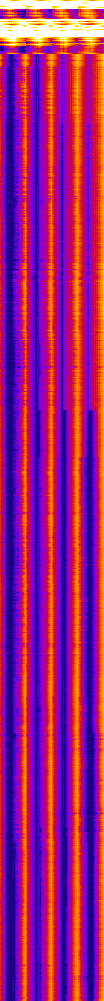

Supplement: Supplementary file 1 [file mmc1.zip › Supplementary-Data/S23-28/Fig.S28-GM-ABCD-2-GM-output_matrix.png]

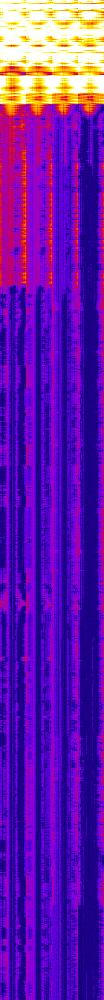

Supplement: Supplementary file 1 [file mmc1.zip › Supplementary-Data/S23-28/Fig.S27-GM-ABCD-1-GM-output_matrix.png]

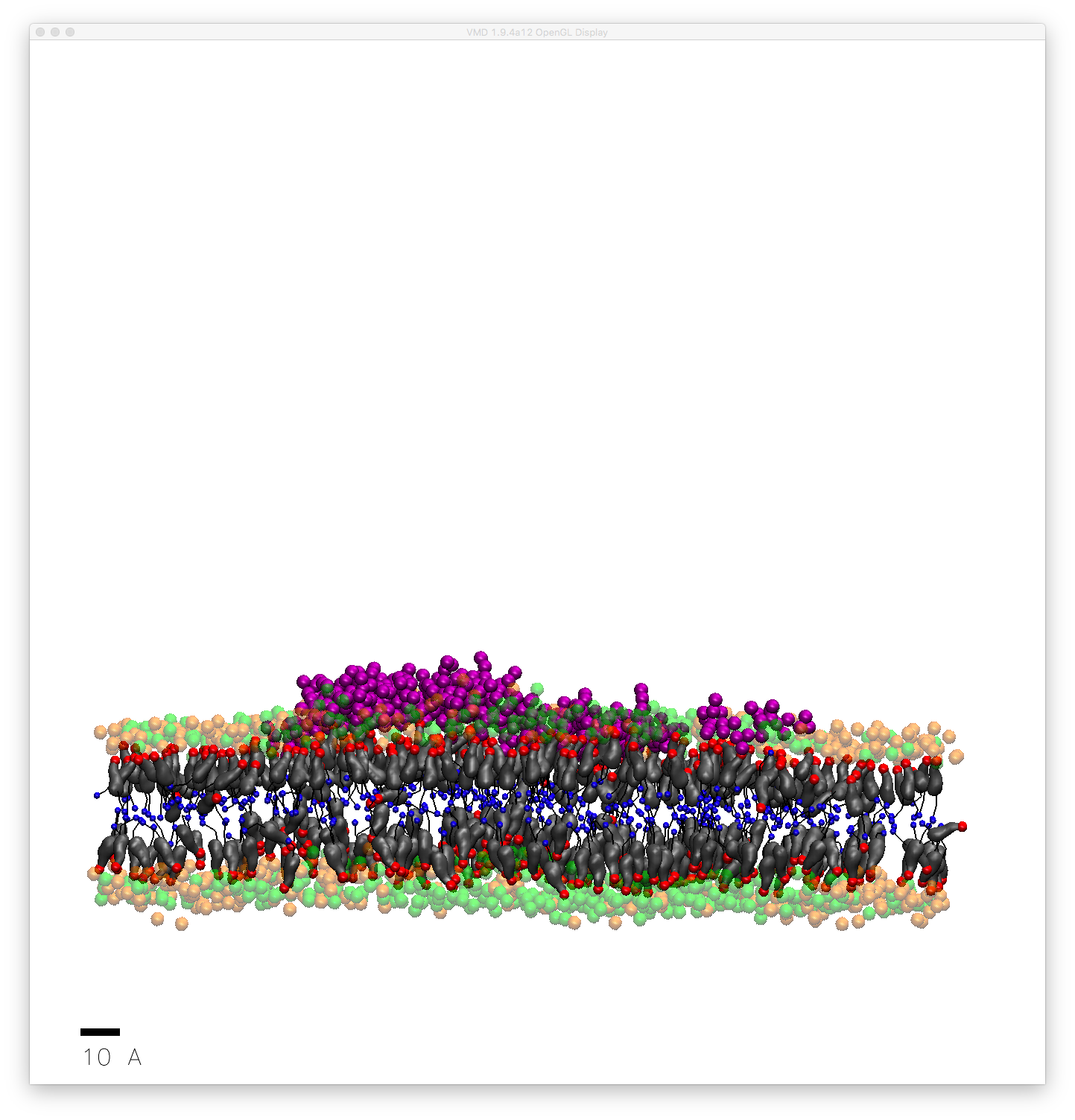

Supplement: Supplementary file 1 [file mmc1.zip › Supplementary-Data/S04/Figure-S4B-GM-raft.png]

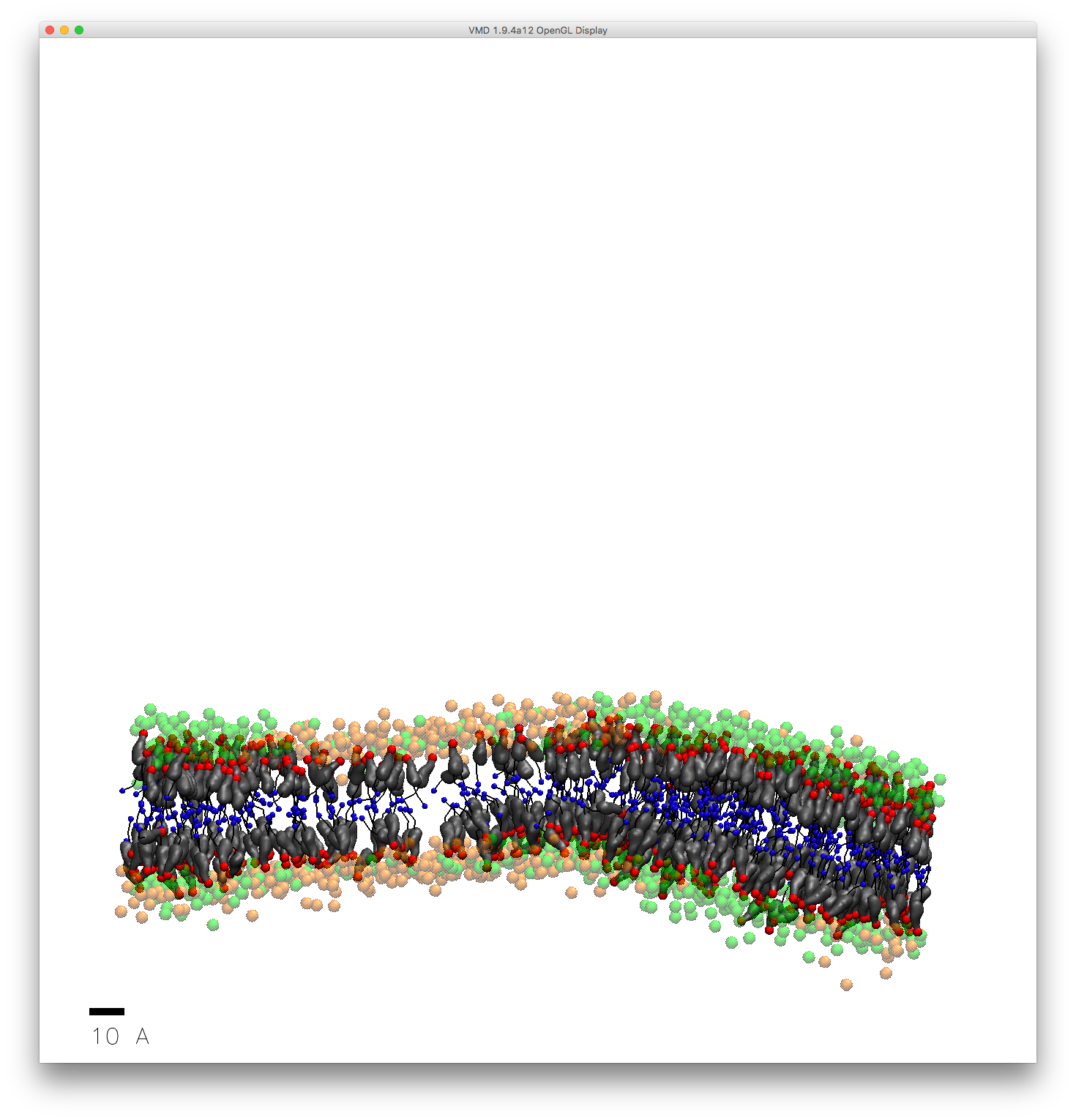

Supplement: Supplementary file 1 [file mmc1.zip › Supplementary-Data/S04/Figure-S4A-P4-raft.png]

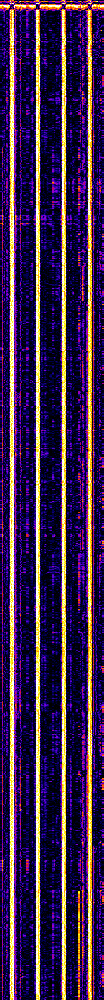

Supplement: Supplementary file 1 [file mmc1.zip › Supplementary-Data/S13-14/Figure-S14C-GM-ABCD-1.png]

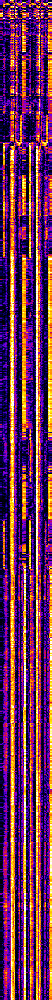

Supplement: Supplementary file 1 [file mmc1.zip › Supplementary-Data/S13-14/Figure-S14B-GM-AB-2.png]

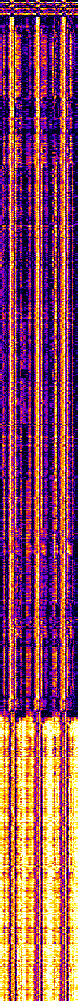

Supplement: Supplementary file 1 [file mmc1.zip › Supplementary-Data/S13-14/Figure-S13D-P1-ABC-2.png]

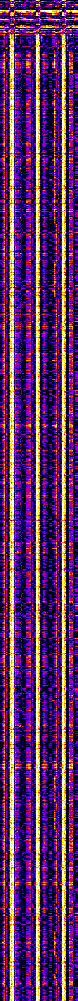

Supplement: Supplementary file 1 [file mmc1.zip › Supplementary-Data/S13-14/Figure-S13C-C1-ABC-3.png]

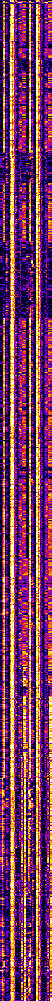

Supplement: Supplementary file 1 [file mmc1.zip › Supplementary-Data/S13-14/Figure-S14A-GM-AB-3.png]

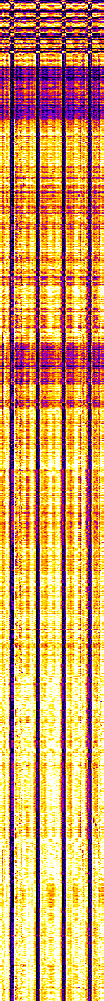

Supplement: Supplementary file 1 [file mmc1.zip › Supplementary-Data/S13-14/Figure-S14D-GM-ABCD-2.png]

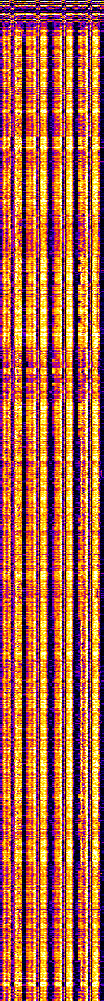

Supplement: Supplementary file 1 [file mmc1.zip › Supplementary-Data/S13-14/Figure-S13B-CO-ABCD-3.png]

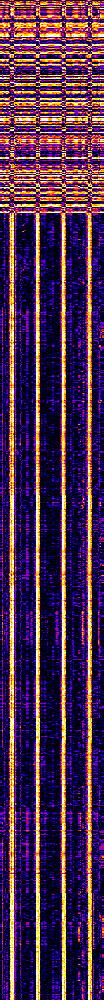

Supplement: Supplementary file 1 [file mmc1.zip › Supplementary-Data/S13-14/Figure-S13A-CO-ABCD-2.png]

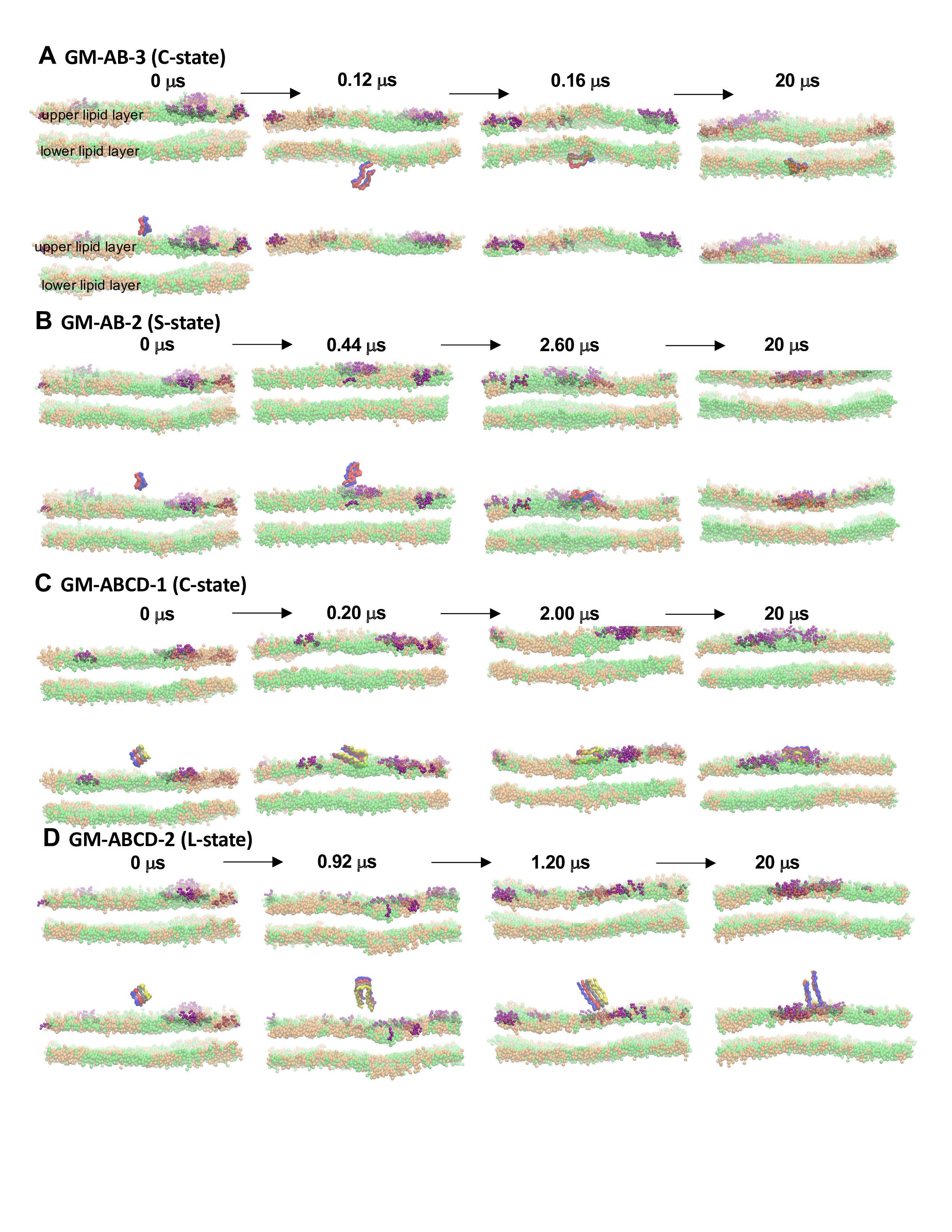

Supplement: Supplementary file 1 [file mmc1.zip › Supplementary-Data/S06-7/Figure-S7.jpg]

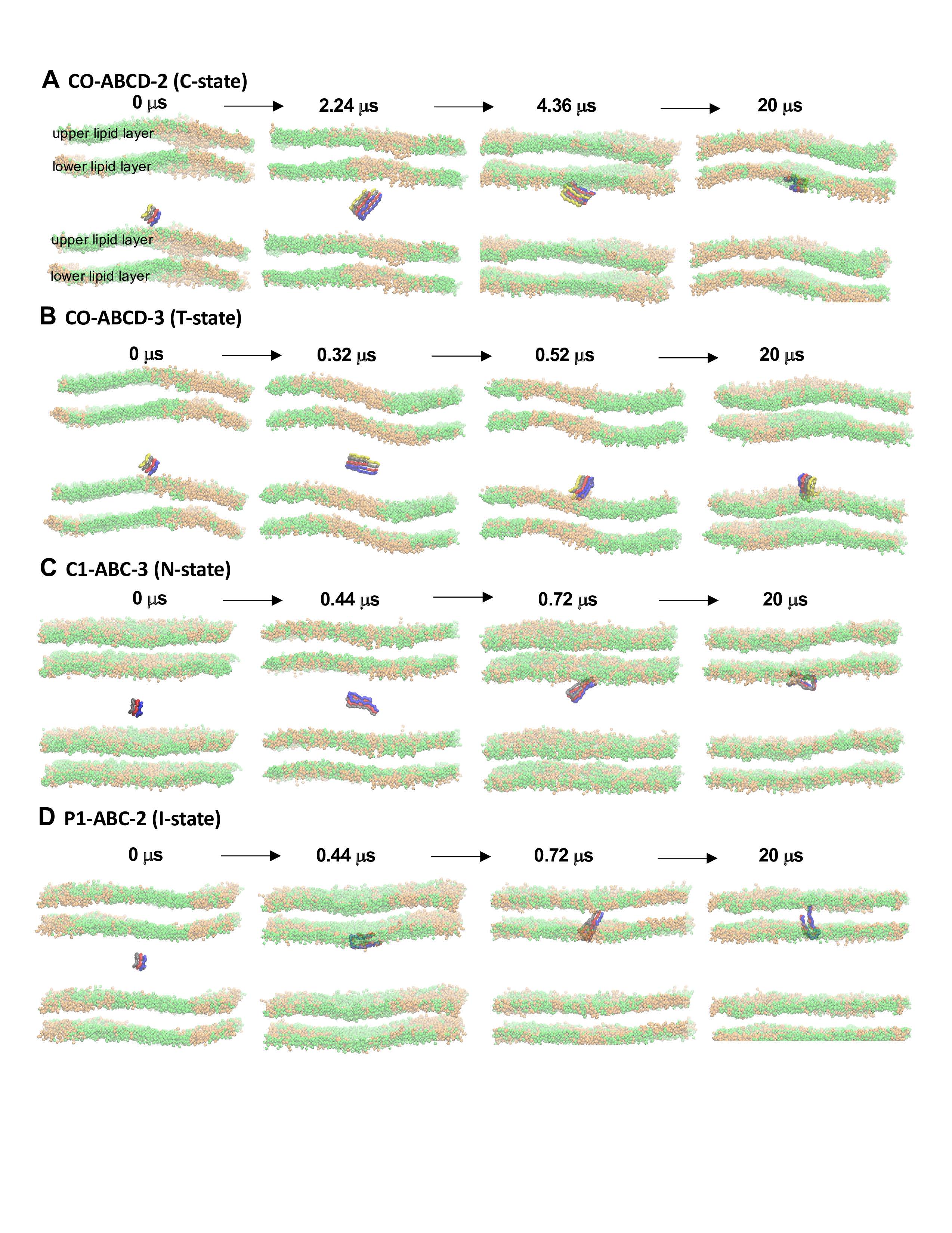

Supplement: Supplementary file 1 [file mmc1.zip › Supplementary-Data/S06-7/Figure-S6.jpg]
